# The Time-Evolution of States in Quantum Mechanics according to the *ETH*-Approach

**DOI:** 10.1007/s00220-021-04225-5

**Published:** 2021-10-23

**Authors:** Jürg Fröhlich, Alessandro Pizzo

**Affiliations:** 1 Institut für theoretische Physik, ETH-Zürich, Zurich, Switzerland; 2 Dipartimento di Matematica, Università di Roma “Tor Vergata”, Rome, Italy

## Abstract

It is argued that the Schrödinger equation does not yield a correct description of the quantum-mechanical time evolution of states of isolated physical systems featuring events. A general statistical law replacing unitary Schrödinger evolution of states is then formulated within the so-called *ETH*-Approach to Quantum Mechanics. This law eliminates the infamous “measurement problem.” Our general concepts and results are illustrated by an analysis of simple models describing a very heavy atom coupled to the quantized radiation field. In the limit where the speed of light tends to infinity these models can be treated quite explicitly.

## Introduction: In Search of a New Law of Nature

“*... their attempts to see in the very inadequacy of the conventional interpretation of quantum theory a deep physical principle have often led physicists to adopt obscurantist, mystical, positivist, psychical, and other irrational worldviews.”* (David Deutsch [1])

In this paper we attempt to add a *missing piece* that has long been searched for to the puzzle of Quantum Mechanics (henceforth abbreviated as QM), namely an appropriate notion of states^1^ and a general **statistical law** governing the *time evolution of states* of isolated physical systems featuring events, (i.e., of what we call “isolated, but *open* systems”). Along the way we intend to dispose of the misconception that unitary Schrödinger evolution of unit rays in Hilbert space, and of density matrices describing mixed states, provides that missing piece.

Disagreement concerning the right notion of states in QM and the nature of a general law describing their time evolution has persisted for almost a century, despite various proposals of how to resolve it; see, e.g., [2–8] and references given there. This has perpetuated a never ending debate about the deeper meaning of QM and has caused a lot of confusion—as deplored by *David Deutsch*. Indeed, *Sean Carroll* has expressed the following pessimistic assessment of the present level of understanding Quantum Mechanics: *“What we don’t do is claim to* $\mathbf {understand}$ *quantum mechanics. Physicists don’t understand their own theory any better than a typical smartphone user understands what’s going on inside the device.”* (Sean Carroll, in: New York Times 2019)

But the problem is *not* that we may not have understood the deeper meaning of QM—in other words that we may not have found the correct interpretation of QM, yet. The problem is that we have *not* accomplished a *complete formulation of the theory called Quantum Mechanics*, yet, as pointed out, e.g., by *Paul Adrien Maurice Dirac* (quoted below)! Perhaps, this is also what *Richard Feynman* may have vaguely had in mind when he said: *“I cannot define the real problem; therefore I suspect there’s no real problem; but I’m not sure there’s no real problem.”*—We actually think there *is* a *real problem*, and the present state of affairs in our comprehension of Quantum Theory is really most unsatisfactory, indeed, and should be changed for the better, as soon as possible!

The main purpose of this paper is not only to formulate a general law describing the evolution of states in non-relativistic QM, but to exemplify it by analyzing a class of simple models of a very heavy atom coupled to the radiation field (in a limit where the speed of light tends to $\infty$), building on ideas described in [9–12]. Although a superior theory, as compared to non-relativistic QM, *local relativistic quantum theory* is technically more complicated; and we do not know any four-dimensional models of this theory with non-trivial interactions that have been shown to be mathematically consistent. The relativistic theory has been considered in [13] and will be studied in more detail in forthcoming work.

Here is a metaphor for the present situation and the task to be accomplished: Until now, QM rests on only three pillars, to be recalled in the next section, and its foundations remain shaky. Our task is to construct a *Fourth Pillar*^2^ that will render the foundations of QM complete and stable. Built into our approach towards accomplishing this task is the fundamental dichotomy of *future*, as the realm of potentialities, versus *past*, as the realm of actualities and facts. It thus incorporates *Aristotle’*s distinction between potentialities and actualities.

One of the basic insights underlying our approach is that, while, in the Heisenberg picture, the time evolution of operators representing physical quantities of an isolated system continues to be described by conjugation with its unitary propagator (Heisenberg time evolution), the physical states of the system are *not* constant in time, but evolve *stochastically*. The fact that, in the Heisenberg picture, a non-trivial time evolution of states turns out to be compatible with the standard deterministic Heisenberg evolution of operators representing physical quantities is derived as a consequence of a fundamental principle—called the *Principle of Diminishing Potentialities*—which says that potentialities become fewer, as time increases. As in the relativity principle, massless modes might play a crucial role in substantiating our pinciple.

Next, we describe the main ideas underlying our approach to Quantum Mechanics by summarizing the contents of this paper.

In Sect. 2, some essential but standard elements of Quantum Mechanics are reviewed: As usual, we represent physical quantities characteristic of a physical system by self-adjoint operators acting on a separable Hilbert space. The Heisenberg picture and the usual Heisenberg equations^3^ for the time evolution of operators representing physical quantities of *isolated* systems are described. (We remark that, for a system interacting with its environment, the time evolution of operators representing physical quantities of that system is *not* given by the usual Heisenberg equations and, because of the influence of the environment on the system, can be arbitrarily complicated. This motivates us to limit our general analysis of the quantum-mechanical time evolution of operators and states to *isolated* systems.) We emphasize the important property that, in the Heisenberg picture, potentially measureable physical quantities are localizable in bounded intervals of the time axis.

The Copenhagen Interpretation of Quantum Mechanics, including the “state reduction-, or collapse postulate,” is briefly recapitulated, criticized and put in perspective.

Section 3 is devoted to a short summary of the so-called *ETH*-Approach to Quantum Mechanics, (where “*ETH*” stands for “*Events, Trees* and *Histories*”). This approach has been developed with the intention to provide a completion of Quantum Mechanics that gets rid of conundrums such as the so-called “measurement problem.” Further details concerning the *ETH*-Approach can be found in earlier papers; see [9–14]. Precise notions of *potential events/potentialities* and of *actual events/actualities* are introduced; (for earlier work introducing various notions of “events” in QM, see [15–18] and references given there). Our notions of *potentialities* and *actualities* reflect the fundamental dichotomy of *future* and *past*. A physically meaningful concept of states of isolated physical systems is proposed. We then describe the so-called *Principle of Diminishing Potentialities* and offer a concise formulation of the *state-reduction-*, or *collapse postulate* (Axiom CP). These ingredients enable us to formulate a general *statistical law* describing the time evolution of states of isolated physical systems.

The *Principle of Diminishing Potentialities* says, roughly speaking, that the algebra generated by all operators describing potentialities possibly occurring at some time $t'$ or later is *properly contained* in the algebra generated by all the operators encoding potentialities that might occur at a time *t* or later, with $t<t'$. It turns out that this Principle can be understood to be a consequence of *Huygens’ Principle* in local relativistic quantum theories with massless particles, which has been analyzed and used in interesting work by *Detlev Buchholz* and *John Roberts*; see [19, 20]. (The *only* result in their joint work relevant for the present paper is one that says that, in a local relativistic quantum field theory with massless modes on Minkowski space $\mathbb {M}^{4}$, such as *quantum electrodynamics*, the algebra generated by all operators localized in the *future* of a space-time point *P* *strictly shrinks*, as *P* moves into the future along a time-like curve.)

In Sect. 4, the connection between *Huygens’ Principle* and the *Principle of Diminishing Potentialities* is recalled in the example of the free quantized electromagnetic field.^4^ We then introduce a simple semi-relativistic model (with discrete time) illustrating the *ETH*-Approach and, in particular, the *Principle of Diminishing Potentialities*. We also comment on the form this principle takes in the limit where the speed of light tends to infinity. In this form it is profoundly relevant for the analysis of models of non-relativtistic Quantum Mechanics, such as the ones studied in Sect. 5.

The most novel section of this paper is Sect. 5. It is devoted to a rather detailed study of non-relativistic quantum-mechanical models illustrating the *ETH*-Approach. These models can be interpreted as describing a very heavy atom with a finite-dimensional Hilbert space of internal states coupled to a caricature of the quantized electromagnetic field, called *R-field*, arising in the limit of the speed of light tending to infinity. The models are chosen so as to minimize technical complexity, but not to loose essential aspects of the *ETH*-Approach. Time is chosen to be discrete, and operators representing physical quantities localized in subsets of the discrete time axis, $\mathbb {Z}$, containing only finitely many times generate *finite-dimensional* matrix algebras. For these models, an explicit law for the time evolution of states is derived, assuming that the *R*-field is prepared in a state *“without memory”*. In this special situation, the resulting effective time evolution of the state of the atom turns out to be “Markovian.” This is however *not* the case if the *R*-field is prepared in a state entangling modes localized at different times.

We then discuss some key implications of our law for the time evolution of states in two limiting regimes: a regime where the atom is only very weakly coupled to the *R*-field and, as a consequence, linear unitary *Schrödinger evolution* is a good approximation to the true evolution of states, except for rare *“quantum jumps”*; and a regime where the degrees of freedom of the atom are very strongly coupled to the degrees of freedom of the *R*-field and, as a consequence, the evolution of states is well approximated by a *classical Markov chain*. The section concludes with an explanation of how, in these models, *“projective measurements”* can be described in a very natural way.

In Sect. 6, we comment on the *ontology* that underlies a quantum-mechanical description of Nature according to the *ETH*-Approach. We then sketch how the models discussed in Sect. 5 can be extended to non-relativistic models with a *continuous* time. We observe that, in such models, the spectrum of the Hamiltonian is unbounded from above *and* below. Finally, we comment on the problem of understanding whether there are alternatives to Huygens’ Principle in deriving the Principle of Diminishing Potentialities.

A tantalizing conclusion of our analysis is that a quantum theory based on a classical flat space-time satisfying this principle, as well as the *spectrum condition*, which says that the energy spectrum of the Hamiltonian of the theory must be bounded from below (i.e., contained in a half-bounded interval of the real line), appears to be necessarily a *local relativistic quantum theory*.

*Remark*: In this paper we do not review the quantum theory of indirect (weak) measurements, which is well developed, taking certain results in a theory of direct (projective) measurements and events for granted. See [21, 22] for recent results and plenty of references.

## Three of the Four Pillars Quantum Mechanics Rests Upon

*“It seems clear that the present quantum mechanics is not in its final form.”* (Paul Adrien Maurice Dirac)^5^

In this section we summarize a few well known basic facts about *non-relativistic Quantum Mechanics*, focussing on the quantum-mechanical description of physical quantities characteristic of a physical system and their dynamics in the *Heisenberg picture*.

Since we consider non-relativistic quantum mechanics, with gravity turned off (or treated as an instantaneous interaction between particles, as conceived by Newton), we may assume that the concept of an *absolute time*, $t\in \mathbb {R}$, parametrising evolution is meaningful; (see [13] for a sketch of a *space-time* approach to local relativistic quantum theory).

### The usual three pillars

*“If you are receptive and humble, mathematics will lead you by the hand.”* (Paul Adrien Maurice Dirac)

In this subsection we recall some well known elements (or “pillars”) of a quantum-mechanical description of Nature.

*Pillar 1* *Physical quantities characteristic of a system.*

In quantum mechanics, a physical system, *S*, is characterized by a list of abstract self-adjoint operators,

1 $$\begin{aligned} \mathcal {O}_{S}= \big \{\hat{X}_i = \hat{X}^{*}_i\,\vert \, i \in \mathfrak {I}_S\big \}\,, \end{aligned}$$

with $\mathfrak {I}_S$ a set of indices depending on *S*, where every operator $\hat{X} \in \mathcal {O}_S$ represents a physical quantity characteristic of *S*, such as the total momentum, energy or spin of all particles localized in a specified bounded region of physical space and belonging to an ensemble of (possibly infinitely many) particles constituting the system *S*.^6^ It is assumed that if $\hat{X} \in \mathcal {O}_S$ and *F* is a real-valued, bounded continuous function on the real line then $F(\hat{X})$ belongs to $\mathcal {O}_S$, too. Apart from that, $\mathcal {O}_S$ does not have any interesting structure. It is usually not a (real) linear space, let alone an algebra.

We assume that, at every time *t*, there is a representation of $\mathcal {O}_S$ by selfadjoint operators acting on a separable Hilbert space $\mathcal {H}_S$:

2 $$\begin{aligned} \mathcal {O}_S \ni \hat{X} \mapsto X(t)= X(t)^{*} \in B(\mathcal {H}_S)\,, \end{aligned}$$

where $B(\mathcal {H}_S)$ is the algebra of all bounded operators acting on $\mathcal {H}_S$. Usually, a physical quantity $\hat{X}\in \mathcal {O}_S$ can be localized in space and in time (*Haag* [23] speaks of “local observables,” *Bell* [6, 7] of “local beables”). It can be constructed by testing some hermitian operator-valued density, $\hat{\mathfrak {x}}(\mathbf {x},\tau )$, on space-time, such as a mass-, momentum-, energy- or spin density of a quantum gas, with a real-valued test function $h(\mathbf {x},\tau )$, yielding a self-adjoint operator:

3 $$\begin{aligned} \hat{X}= & {} F\Big [\int d^{3}x\int d\tau \, h(\mathbf {x},\tau )\, \hat{\mathfrak {x}}(\mathbf {x},\tau )\,\Big ]\, \mapsto \nonumber \\ \,\, X(t):= & {} F\Big [ \int d^{3}x\int d\tau \,h(\mathbf {x},\tau )\, \mathfrak {x}(\mathbf {x}, \tau + t)\,\Big ],\,\, t\in \mathbb {R}, \end{aligned}$$

where *F* is an arbitrary real-valued, bounded continuous function on $\mathbb {R}$, and $\mathfrak {x}(\mathbf {x},t+\tau )$ is an operator-valued distribution (acting on $\mathcal {H}_S$) representing the abstract density $\hat{\mathfrak {x}}(\mathbf {x}, \tau )$ at time *t*. Assuming that we only consider test functions *h* with compact support in the time direction, we conclude that the operator *X*(*t*) is localized in a time-slice, $I\times \mathbb {E}^{3}$, of finite width, where $I\equiv I_{X(t)}$ is a bounded interval of the time axis (assumed to contain the time *t* in its interior), and $\mathbb {E}^{3}$ is physical space.

*Pillar 2* *Heisenberg-picture dynamics of operators.*

Next, we recall how the time evolution of physical quantities in the *Heisenberg picture* is described. For this purpose we have to introduce the notion of an **isolated (physical) system**. An isolated system *S* is one whose degrees of freedom have negligibly weak interactions with the degrees of freedom of its complement, $S^{c}$, i.e., with the rest of the Universe, during the period in time when the evolution of *S* is monitored. (Yet, the state of $S\vee S^{c}$ can be entangled!) As discovered by *Heisenberg* and *Dirac*, it is *only* for an *isolated* system, *S*, that we are able to formulate a general dynamical law for the time evolution of physical quantities characteristic of *S*. For simplicity, we temporarily assume that the system *S* is *autonomous*. Then there exists a selfadjoint operator, $H_S \equiv H=H^{*}$, acting on the Hilbert space $\mathcal {H}_S$, the *Hamiltonian* of the system *S*, such that the concrete self-adjoint operators on $\mathcal {H}_{S}$ representing some physical quantity $\hat{X}\in \mathcal {O}_S$ at two different times, *t* and $t'$, are unitarily conjugated to each other by the propagator generated by *H*, i.e.,

4 $$\begin{aligned} X(t') = e^{i(t'-t)H} \,X(t) \,e^{-i(t'-t)H}, \quad \text {for arbitrary times }\,\,\, t, t'\,, \end{aligned}$$

where *X*(*t*) represents $\hat{X}$ at time *t* (see Eq. (2)). This equation is commonly referred to as the *Heisenberg equation*. It encapsulates the *deterministic* law of time evolution of operators on $\mathcal {H}_S$ representing physical quantities in $\mathcal {O}_S$ characteristic of the system *S*. Notice that if *X*(*t*) is localized in the time interval $I_{X(t)}$ then $X(t')$ is localized in the interval $I_{X(t')}= I_{X(t)} +(t'-t)$.

Equation (4) is usually extended to arbitrary bounded operators on $\mathcal {H}_S$:

5 $$\begin{aligned} A(t) = e^{itH} \, A \, e^{-itH}\,, \quad \forall A \in B(\mathcal {H}_S)\,, \end{aligned}$$

for arbitrary times $t \in \mathbb {R}$. It is straightforward to extend Eqs. (4) and (5) to non-autonomous isolated systems, whose Hamiltonians are time-dependent.

#### Remark

If there are substantial interactions between the degrees of freedom of *S* and degrees of freedom describing the “environment”, $S^{c}$, of *S* the description of the time evolution of physical quantities characteristic of *S*, i.e., of operators representing elements of $\mathcal {O}_S$, can be arbitrarily complicated. (A description of the dynamics of systems interacting with their environment in terms of, for example, quantum Markov semi-groups generated by Lindblad operators is an approximation that, most of the time, is not justified. However, since it may be adequate, qualitatively, it is widely used.)

*Pillar 3* *Expectation values of physical quantities in “states.”*

In order to extract concrete information about the behavior of (an ensemble of identical) isolated physical systems, *S*, as described in Pillars 1 and 2, one has to be able to take expectation values of self-adjoint operators *X*(*t*) on $\mathcal {H}_S$ representing physical quantities $\hat{X} \in \mathcal {O}_S$. For this purpose, one introduces some notion of *“state”*. In non-relativistic quantum mechanics, “states” are usually taken to be *density matrices* on $\mathcal {H}_S$, which are non-negative, trace-class operators, $\widetilde{\Omega }$, acting on $\mathcal {H}_S$ of trace 1, i.e.,

6 $$\begin{aligned} \widetilde{\Omega } = \widetilde{\Omega }^{*} \ge 0,\,\,\text{ with } \,\, \text {tr}(\widetilde{\Omega }) =1\,. \end{aligned}$$

(In the following, we usually refer to these states as *normal states*. For all possibly unfamiliar notions concerning abstract functional analysis see, e.g., [24, 25].) Pure states are given by orthogonal projections, $P=P^{*}=P^{2}$, of rank 1 corresponding to unit rays in $\mathcal {H}_S$. The expectation, $\omega (X(t))$, of a physical quantity, $\hat{X}\in \mathcal {O}_S$, at time *t* in a “state” given by a density matrix $\widetilde{\Omega }$ is defined by

7 $$\begin{aligned} \omega (X(t)):= \text {tr}(\widetilde{\Omega }\cdot X(t))\,, \end{aligned}$$

where *X*(*t*) represents $\hat{X}$ at time *t*. Equation (7) is then extended to arbitrary bounded operators on $\mathcal {H}_S$, i.e.,

$$\begin{aligned} \omega (A):= \text {tr}(\widetilde{\Omega }\cdot A), \qquad \forall A \in B(\mathcal {H}_S)\,. \end{aligned}$$

We will see shortly that this notion of “state” does not have any ontological meaning.^7^

It is common to claim that, in the Heisenberg picture, only operators evolve non-trivially in time, but states $\omega$ are *time-independent*. One then usually goes on to claim that the Heisenberg picture is equivalent to the *Schrödinger picture*, where physical quantities are *time-independent* but “states” evolve in time according to the *Schrödinger-Liouville equation*

8 $$\begin{aligned} \Omega (t)= e^{-i(t-t')H} \,\Omega (t') \, e^{i(t-t')H}\,, \quad \text {for arbitrary times }\,\, t, t'\,, \, \text { with }\, \Omega (0)\equiv \widetilde{\Omega }\,. \end{aligned}$$

One then obviously has that

$$\begin{aligned} \text {tr}\big (\widetilde{\Omega }\cdot A(t)\big )= \text {tr}\big (\Omega (t)\cdot A\big ), \qquad \forall A \in B(\mathcal {H}_S)\,, \end{aligned}$$

see Eqs. (5), (8).

#### Remark

In the following, we use a “tilde” to indicate that we refer to a density matrix in the Heisenberg picture, while we drop the “tilde” in the Schrödinger picture. We usually identify a density matrix in the Heisenberg picture with the corresponding density matrix in the Schrödinger picture at time $t=0$.

### Measurements and the collapse postulate

*“The concept of ‘measurement’ becomes so fuzzy on reflection that it is quite surprising to have it appearing in physical theory ...”* (John Stewart Bell)

We note that Eqs. (4), (5) and (8) are **linear** and **deterministic** evolution equations. However, most physicists agree with the claim that the predictions of QM are **statistical** (probabilistic). So, what is going on? Well, according to the *Copenhagen Interpretation of QM*, the Schrödinger-Liouville evolution of states given by Eq. (8) is interrupted whenever *“a measurement takes place,”* and in such a moment the Schrödinger-Liouville evolution is replaced by a non-linear change of state described—at least heuristically—by the so-called *state-reduction-, or collapse postulate*: If a physical quantity $\hat{X}$ is measured at some time *t*, with the outcome that it has a measured value $\xi$ belonging to the spectrum, $\text {spec}(\hat{X})$, of the operator $\hat{X}$, then the state, $\widetilde{\Omega }$, occupied by the system *S* right *before* the measurement of $\hat{X}$ is carried out is supposed to be replaced by the state, $\widetilde{\Omega }_{\xi , t}$, given by

9 $$\begin{aligned} \widetilde{\Omega } \,\mapsto \, \widetilde{\Omega }_{\xi , t} := \big [\text {tr}\big (\widetilde{\Omega }\,\Pi _{\xi }(t)\big )\big ]^{-1}\, \Pi _{\xi }(t)\, \widetilde{\Omega }\, \Pi _{\xi }(t)\,, \end{aligned}$$

right *after* the measurement of $\hat{X}$, where $\Pi _{\xi }(t)$ is the spectral projection corresponding to the eigenvalue $\xi$ of the self-adjoint operator *X*(*t*) representing $\hat{X}$ at time *t*; and the probability of measuring the value $\xi$ is given by $\text {tr}\big (\widetilde{\Omega }\,\Pi _{\xi }(t)\big )$—*Born’s Rule*.

The question then arises what the precise quantum-mechanical law is that determines under what conditions a “measurement” takes place (is carried out), and at what time the state-collapse (9) resulting from the measurement happens. Answering this question will amount to adding a *“Fourth Pillar”* to the formulation of QM. Actually, the prescription in Eq. (9) is *at best* a reasonable heuristic recipe, but does obviously not have the status of a general law, as long as the notion of “measurement” remains totally vague and does not correspond to a well-defined operation in the mathematical formalism of QM, and as long as the time when a measurement happens cannot be predicted by the theory!^8^

### Inadequacy of unitary Schrödinger evolution of states of physical systems

*“I insist upon the view that ‘all is waves’.”* (Erwin Schrödinger, letter to J.L. Synge)

Before we will describe a precise general law governing the quantum-mechanical time evolution of states of isolated systems (see Sects. 3, 4, 5) we stress that we do *not* consider unitary Schrödinger evolution to provide such a law—*even* if all the experimental equipment used to perform measurements on a given sub-system of interest is included in the quantum-mechanical description (so that the resulting *total* system is *isolated*). This view has already been emphasized by some of the founding fathers of Quantum Mechanics; (see Heisenberg’s lucid discussion in [26]^9^ and refs. given there). An interesting argument showing that nothing but unitary Schrödinger evolution of states of isolated systems leads to paradoxes is the so-called *“Wigner’s friend paradox”*. Rather than recalling it here, we refer the reader to the original paper [27], and to [28, 29] for more recent variants of the original argument. In [14], an alternative argument showing that unitary Schrödinger evolution of states of isolated systems is often incompatible with what is observed in experiments has been described. This argument is based on a quantitative analysis of correlations between the outcomes of measurements of physical quantities (spins) referring to two different sub-systems that have been prepared in an entangled state. The analysis in [14] also illustrates what people call the *“non-locality”* of Quantum Mechanics; for a recent study, see, e.g., [30]. (For a discussion of the crucial role of *locality* in relativistic quantum theory, in the sense of Einstein causality, as opposed to the “non-locality” just referred to, the reader may consult [13, 23].)

To conclude this section, we remark that we do not consider the so-called *relative state formulation*, or *many-worlds interpretation*, of Quantum Mechanics, due to Everett [4, 5], as a valid justification of the idea that *unitary Schrödinger evolution*, amended by some rules for “branching” in the evolution of the state of an isolated system, of which a precise formulation appears to be lacking, may lead to a satisfactory description of reality. Everett’s formalism does not appear to provide a logically coherent formulation of Quantum Mechanics apt to eliminate persistent difficulties, such as the “measurement problem,” and to correctly describe the emergence of events and the outcome of experiments.

We do not wish to further discuss the thorny subject of *“Interpretations of Quantum Mechanics.”*

## The Fourth Pillar of Quantum Mechanics—Summary of the $\mathbf {\textit{ETH}}$-Approach

“*Surely, after 62 years, we should have an exact formulation of some serious part of quantum mechanics. ... By ‘serious’ I mean that some substantial fragment of physics should be covered.”* (John Stewart Bell)

In this section we endeavor to sketch a pragmatic formulation of QM, the *ETH-Approach*, which is intended to eliminate those undesirable worldviews David Deutsch has been referring to in [1]. In particular, it is intended to replace *“interpretations”* of QM by a *completion* of QM freed from puzzles such as the *“measurement problem.”* We view the *ETH*-Approach to QM as representing the *Fourth Pillar* QM rests upon. It is expected to provide stable foundations to the theory.

The scope of this paper is limited to non-relativistic QM; see [10, 11]. The general ideas underlying the *ETH*-Approach can be extended to local relativistic quantum theory, but the analysis becomes more subtle; for a beginning see [13].

### Algebras of potentialities and quantum probability measures

*“It is not the past that matters but the future.”* (Varun Ravikumar)

From Sect. 2 we recall that operators, *X*(*t*), representing physical quantities, $\hat{X}$, characteristic of a system *S* at some time *t* are self-adjoint operators, i.e., $X(t)=X(t)^{*}$, acting on a separable Hilbert space $\mathcal {H}_S$. As argued in Sect. 2, one can associate a bounded interval $I_{X(t)}$ of the time axis containing *t* with every such operator *X*(*t*); see Eqs. (2), (3) and (4). It is natural to introduce algebras, $\mathcal {E}_I\,, I \subset \mathbb {R}$, as the algebras generated by arbitrary complex linear combinations of arbitrary products of operators *X*(*t*) representing physical quantities $\hat{X} \in \mathcal {O}_S$ (see Eqs. (1), (2)), with the property that $I_{X(t)} \subseteq I$, and of the identity, $\mathbf {1}$, on $\mathcal {H}_S$; (the identity $\mathbf {1}$ belongs to *all* the algebras $\mathcal {E}_I$). We then define algebras $\mathcal {E}_{\ge t}$ as follows.

10 $$\begin{aligned} \mathcal {E}_{\ge t} := \overline{\bigvee _{I \subset [t, \infty )} \mathcal {E}_I}\,, \end{aligned}$$

where (to be specific) the closure on the right side is taken in the topology of weak convergence on $\mathcal {H}_S$. The algebra $\mathcal {E}_{\ge t}$ is called the *algebra of all potentialities at times* $\ge t$. (It is a von Neumann algebra.) It follows directly from the definition that

11 $$\begin{aligned} \mathcal {E}_{\ge t'} \subseteq \mathcal {E}_{\ge t}\,, \quad \forall t' > t\,. \end{aligned}$$

We also define $\mathcal {E}$ to be the norm-closure of the algebra generated by $\big \{ \mathcal {E}_{\ge t} \big \}_{t\in \mathbb {R}}$; ($\mathcal {E}$ is the algebra of all potentialities in the history of the system *S*).

#### Remark

Let *S* be an isolated autonomous system with Hamiltonian *H*, and let $t'>t$. Then Eq. (4) for the time evolution of operators in the Heisenberg picture implies that

12 $$\begin{aligned} \mathcal {E}_{\ge t'}= \big \{e^{i(t'-t)H} X\, e^{-i(t'-t)H} \,\vert \, X\in \mathcal {E}_{\ge t}\big \}\,\subseteq \mathcal {E}_{\ge t}\,, \end{aligned}$$

i.e., time evolution of operators in the Heisenberg picture by an amount $t'-t>0$ determines a $^{*}$*endomorphism* of $\mathcal {E}_{\ge t}$ whose image is the algebra $\mathcal {E}_{\ge t'}$. It turns out that this important feature distinguishes the *ETH*-Approach from various rather vague schemes based on the observation that time evolution of a system may entangle its degrees of freedom with those of an unobserved or unobservable environment. (We will come back to this point in Sect. 6)

#### Definition 1

*(Potentialities)*. A *potential event* or *potentiality* associated with the system *S* that might set in at time *t* (i.e., is localized at times $\ge t$) is given by a partition of unity by orthogonal projections on $\mathcal {H}_S$,

$$\begin{aligned} \big \{ \pi _{\xi }\, \vert \,\xi \in \mathfrak {X} \big \} \subset \mathcal {E}_{\ge t}\,, \end{aligned}$$

where $\mathfrak {X}$ is a countable set, with the following properties:

13 $$\begin{aligned} \quad \pi _{\xi }= \pi _{\xi }^{*} \in \mathcal {E}_{\ge t}, \quad \forall \xi \in&\mathfrak {X}\,,\quad \pi _{\xi }\cdot \pi _{\eta } = \delta _{\xi \eta }\, \pi _{\xi } \,, \quad \forall \, \xi , \eta \text { in }\, \mathfrak {X}\,, \quad \text { and } \quad \sum _{\xi \in \mathfrak {X}} \pi _{\xi } = \mathbf {1}\,. \end{aligned}$$

$$\square $$

From now on, the reader is invited to think of the algebra $\mathcal {E}_{\ge t}$ as the (von Neumann) algebra generated by all potential events setting in at times $\ge t$, for any $t\in \mathbb {R}$.^10^

Let $\mathcal {P}_{\ge t}$ be the lattice of all orthogonal projections in $\mathcal {E}_{\ge t}$.

#### Definition 2

*(Quantum probabilities)*. A *quantum probability measure* on the potentialities localized at times $\ge t$ is a map $\mu \,: \mathcal {P}_{\ge t} \rightarrow [0, 1]$ with the following properties:

(i) $$0 \le \mu (\pi ) \le 1\,, \forall \, \pi \in \mathcal {P}_{\ge t},$$   with    $$\mu (0)=0\,$$   and   $$\mu (\mathbf {1})=1\,$$;
(ii) $\mu \big (\sum _{\xi \in \mathfrak {X}_0} \pi _{\xi }\big ) = \sum _{\xi \in \mathfrak {X}_0} \mu \big (\pi _{\xi }\big )\,,$  for an arbitrary potentiality $\big \{ \pi _{\xi }\, \vert \, \xi \in \mathfrak {X} \big \} \subset \mathcal {E}_{\ge t}$ and an arbitrary subset $\mathfrak {X}_0 \subseteq \mathfrak {X}$ .

$$\square $$

The following generalization of *Gleason*’s theorem [31] follows directly from a general theorem due to *Maeda* [32].

#### Theorem 1

(Gleason-Maeda). We assume that the algebras $\mathcal {E}_{\ge t}$ do not have any direct summand given by the algebra of all complex $2\times 2$ matrices. Then every probability measure, $\mu$, on the potentialities setting in at time *t* is given by a normal state, $\omega _{\mu }$, on the von Neumann algebra $\mathcal {E}_{\ge t}$, with

$$\begin{aligned} \mu (\pi )= \omega _{\mu }(\pi ),\qquad \forall \pi \in \mathcal {P}_{\ge t}\,. \end{aligned}$$

$$\square $$

(A normal state on a von Neumann algebra $\mathfrak {M}$ is defined to be a positive linear functional, $\omega$, on $\mathfrak {M}$ continuous in the weak topology and normalized such that $\omega (\mathbf {1})=1$.)

#### Remarks

1. If $\big \{ \pi _{\xi }\, \vert \,\xi \in \mathfrak {X} \big \} \subset \mathcal {E}_{\ge t}$ is a potential event localized at times $\ge t$ then $\big \{ e^{isH}\pi _{\xi } e^{-isH} \,\vert \, \xi \in \mathfrak {X} \big \} \in \mathcal {E}_{\ge (t+s)}$ is a potential event localized at times $\ge (t+s)$; (*H* is the Hamiltonian of the system).
2. For autonomous systems, *S*, with *finitely many degrees of freedom*, the algebras $\mathcal {E}_{\ge t}$ coincide with the algebra $B(\mathcal {H}_S)$ of all bounded operators on $\mathcal {H}_S$ and, hence, are *independent* of *t*. It turns out that, for such systems, it is *impossible* to introduce a non-trivial notion of *events actually happening* (actualities) at some time *t* or later, *and the so-called* **measurement problem** *cannot be solved* by considering only such systems. The situation is radically different if one considers systems for which the inclusions in (11) are *strict*, which can happen for systems with infinitely many degrees of freedom including ones describing *massless modes*, such as photons and gravitons, that can escape to infinity, at the limiting speed, *c*, without being detected; see Sect. 4.

#### Definition 3

*(Closed systems)*. A physical system *S* is said to be a *closed system* iff the algebras $\mathcal {E}_{\ge t}$ of all potentialities setting in at time *t* are *independent* of *t*, for all times $t\in \mathbb {R}$ (i.e., equality holds in (11), for all times *t* and $t'$). Closed systems have the same defects as systems with finitely many degrees of freedom: The measurement problem cannot be solved for such systems.

### The Principle of Diminishing Potentialities

*“Indeed, it is evident that the mere passage of time itself is destructive rather than generative ..., because change is primarily a ‘passing away.’ ”* (Aristotle, Physics)

In order to introduce a good notion of *events actually setting in at some time* *t* (for short: actualities) and to clarify how such events can be recorded in *projective measurements*, we require the following

*Principle of Diminishing Potentialities* (*PDP*). An isolated system *S* featuring actualities, i.e., events that set in at some (finite) time, has the property that

14 $$\begin{aligned} \mathcal {E}_{\ge t'} \subsetneqq \mathcal {E}_{\ge t} \subsetneqq \mathcal {E}\,, \quad \text {whenever}\,\,\,\, t'>t\,. \qquad \, (PDP) \end{aligned}$$

$$\square $$

This principle has been introduced and carefully analyzed in [9–13].^11^ Our main concern in this paper is to describe a concrete family of models satisfying (*PDP*); see Sects. 4 and 5 . In the models of Sect. 4, the algebras $\mathcal {E}_{\ge t}$ are associated with physical quantities localized inside future light cones in Minkowski space $\mathbb {M}^{4}$ nested inside one another, and (*PDP*) turns out to be a consequence of the existence of massless modes, e.g., photons, whose dynamics satisfies locality or Einstein causality (*Huygens’ Principle*; for a general analysis see [19, 20]). The models studied in Sect. 5 arise in the limit of the speed of light tending to $\infty$. We expect that, in *all* models of *non-relativistic* Quantum Mechanics with a *continuous time*, (*PDP*) only holds if the spectrum of the Hamiltonian, *H*, of the system is *unbounded above and below*; see Sect. 6. However, as argued in [13], in *local relativistic quantum theory*, (*PDP*) is compatible with the spectrum condition $H\ge 0$.

**Description of isolated open systems.**

#### Definition 4

*(Isolated open systems)*. In Quantum Mechanics, an *isolated, but open system*, *S*, is described in terms of a *co-filtration* (i.e., a decreasing filtration), $\lbrace \mathcal {E}_{\ge t} \rbrace _{t \in \mathbb {R}}$, (or $\lbrace \mathcal {E}_{\ge t} \rbrace _{t \in \mathbb {Z}}$, in case *time* is assumed to be discrete, see Sects. 4, 5), of von Neumann algebras, $\mathcal {E}_{\ge t}$, satisfying property (12) and (*PDP*), Eq. (14), all represented on a common Hilbert space $\mathcal {H}_S$, whose lattices of projections describe *potentialities*. $\square$

Let $\omega$ be a state occupied by *S*, as introduced in Eq. (7) of Sect. 2; (see [33] for an analysis of how to prepare a system in a specific state). The state, $\omega _t$, of *S* at time *t*,

15 $$\begin{aligned} \omega _{\,t}(X):= \omega (X), \qquad \forall X \in \mathcal {E}_{\ge t}, \end{aligned}$$

is defined to be the restriction of the state $\omega$ to the algebra $\mathcal {E}_{\ge t}$. By the Gleason-Maeda theorem, $\omega _{\,t}$ corresponds to a quantum probability measure on $\mathcal {P}_{\ge t}$. The state $\omega _{\,t}$ of *S* at some time *t*, as defined in (15), will usually be a *mixed* state *even* if $\omega$ is a pure state on $\mathcal {E}$. This is a consequence of (*PDP*), Eq. (14), and *entanglement*; (see Sects. 4 and 5 for explicit examples). We should then clarify what we mean by saying that $\omega _{\,t}$ is a *mixed state*, and what the implications of this property for the appearance of *“actual events”* are. We begin by formulating a criterion enabling us to decide whether an actual event sets in at time *t*, assuming that we know which state $\omega _t$ the system *S* occupies at a time immediately before *t*.^12^ More precisely, the criterion formulated below enables us to decide whether, given a state $\omega _t$ on the algebra $\mathcal {E}_{\ge t}$, there exists a potential event localized at times $\ge t$ that describes an *actual event* setting in at time *t*.

#### Definition 5

*(The centralizer of a state, and the center of the centralizer)*. (5.i) Given a $^{*}$-algebra $\mathcal {A}$ and a state $\omega$ on $\mathcal {A}$, the centralizer, $\mathcal {C}_{\omega }(\mathcal {A})$, of the state $\omega$ is the subalgebra of all operators $Y \in \mathcal {A}$ with the property that

$$\begin{aligned} \omega ([Y, X]) =0, \qquad \forall X \in \mathcal {A}, \end{aligned}$$

i.e.,

$$\begin{aligned} \mathcal {C}_{\omega }(\mathcal {A}):=\left\{ Y\in \mathcal {A}\, \vert \,\,\omega ([Y, X]) =0, \,\,\forall X \in \mathcal {A}\right\} . \end{aligned}$$

$$\square $$

We note in passing that the state $\omega$ defines a finite (normalized) trace on its centralizer $\mathcal {C}_{\omega }(\mathcal {A})$. This enables one to classify all those von Neumann algebras that can arise as centralizers of normal states on von Neumann algebras.

(5.ii) The *center* of the centralizer $\mathcal {C}_{\omega }(\mathcal {A})$ of the state $\omega$, denoted by $\mathcal {Z}_{\omega }(\mathcal {A})$, is the abelian subalgebra of $\mathcal {C}_{\omega }(\mathcal {A})$ consisting of all operators that commute with *all* other operators in $\mathcal {C}_{\omega }(\mathcal {A})$, i.e.,

$$\begin{aligned} \mathcal {Z}_{\omega }(\mathcal {A}):= \big \{ Y \in \mathcal {C}_{\omega }(\mathcal {A})| \,\,[Y,X]=0\,,\, \forall X \in \mathcal {C}_{\omega }(\mathcal {A}) \big \}. \end{aligned}$$

$$\square $$

We note that the center, $\mathcal {Z}(\mathcal {A})$, of an algebra $\mathcal {A}$ is contained in $\mathcal {Z}_{\omega }(\mathcal {A})$, for all states $\omega$ on $\mathcal {A}$. After these preparations, we define *actual events/actualities* in a system *S* in the following way.

#### Definition 6

*(Actual events/actualities)*. Let *S* be an isolated open system described by a co-filtration $\big \{ \mathcal {E}_{\ge t} \big \}_{t \in \mathbb {R}}$ of von Neumann algebras. Given a state $\omega _t$ on the algebra $\mathcal {E}_{\ge t}$, an *actual event* corresponding to a potential event described by a partition of unity $\big \{ \pi _{\xi }\,\vert \, \xi \in \mathfrak {X} \big \} \subset \mathcal {E}_{\ge t}$  is setting in at time *t* iff $\mathcal {Z}_{\omega _t}(\mathcal {E}_{\ge t})$ is non-trivial,^13^

16 $$\begin{aligned} \big \{ \pi _{\xi }\,\vert \,\xi \in \mathfrak {X} \big \} \,\,\text {generates }\,\, \mathcal {Z}_{\omega _{\,t}}\big (\mathcal {E}_{\ge t}\big ), \end{aligned}$$

and the *Born probabilities*

17 $$\begin{aligned} \omega _{\,t}(\pi _{\xi _{j}}) \,\, \text{ are } \text{ strictly } \text{ positive }, \text{ for } \text{ points } \,\xi _j \in \mathfrak {X}, \,\, j=1,2, \dots , n\,, \end{aligned}$$

for some $$n\ge 2$$. $$\square $$

According to this definition, for a *potential event* $\big \{\pi _{\xi }\,\vert \, \xi \in \mathfrak {X}\big \}\subset \mathcal {E}_{\ge t}$ to be an *actual event* featured by the system *S* during a time period contained in $[t, \infty )$ it is apparently necessary and sufficient that the projections $\big \{\pi _{\xi }\,\vert \, \xi \in \mathfrak {X}\big \}$ generate the center, $\mathcal {Z}_{\omega _t}(\mathcal {E}_{\ge t})$, of the centralizer of the state $\omega _t$ on the algebra $\mathcal {E}_{\ge t}$.

Next, we propose to analyze the consequences of the statement that, in some isolated open system *S*, an actual event or actuality sets in at time *t*.

### Actual events and the state-reduction-/collapse postulate

*“Every experiment destroys some of the knowledge of the system which was obtained by previous experiments.”* (Werner Heisenberg)

Let $\omega _{\,t}$ be the state of *S* right *before* time *t*. Let us assume that an actual event $\big \{ \pi _{\xi } \,|\, \xi \in \mathfrak {X} \big \}$ generating $\mathcal {Z}_{\omega _t}(\mathcal {E}_{\ge t})$ sets in (i.e., begins to unfold) at time *t*. This implies that

18 $$\begin{aligned} \omega _{\,t}(A)= \sum _{\xi \in \mathfrak {X}} \omega _{\,t}(\pi _{\xi } \,A\, \pi _{\xi }), \qquad \forall A \in \mathcal {E}_{\ge t}\,, \end{aligned}$$

i.e., $\omega _{\,t}$ is an *incoherent superposition* of states in the range of the projections $\pi _{\xi }, \xi \in \mathfrak {X}$; (no off-diagonal terms appear on the right side of Eq. (18)). In other words, the quantum probability measure determined by $\omega _{\,t}$ on the potentialities, $\mathcal {P}_{\ge t}$, at times $\ge t$ is a convex combination of quantum probability measures indexed by the points $\xi \in \mathfrak {X}$ that label the projections of the actual event setting in at time *t*. (In this precise sense, $\omega _t$ is a *mixture* indexed by the points of $\mathfrak {X}$.)

*Pillar 4* In the *ETH*-Approach to QM, the following axiom (see [11]) is required in order to complete the formulation of Quantum Mechanics:

**Axiom CP** (Collapse Postulate). *Let* *S* *be an isolated open system satisfying* (*PDP*). *Let* $\omega _t$ *be the state on the algebra* $\mathcal {E}_{\ge t}$ *right* *before* *time* *t*. *Let* $\big \{ \pi _{\xi } \,|\, \xi \in \mathfrak {X} \big \} \subset \mathcal {Z}_{\omega _t}(\mathcal {E}_{\ge t})$ *be the actual event (actuality) setting in at time* *t*. *Then the state on* $\mathcal {E}_{\ge t}$ *occupied by* *S* *right* *after* *the event has set in is given by*

$$\begin{aligned} \omega _{\,t, \xi _{*}}(\cdot ):=[\omega _{\,t}(\pi _{\xi _{*}})]^{-1}\,\omega _{\,t}\big (\pi _{\xi _{*}} (\cdot ) \pi _{\xi _{*}}\big ) \,, \end{aligned}$$

*for some point* $\xi _{*} \in \mathfrak {X}$ *with* $\omega _{\,t}(\pi _{\xi _{*}})>0$. *The probability for the system* *S* *to be found in the state* $\omega _{\,t,\xi _{*}}$ *right* *after* *time* *t* *is given by* **Born’s Rule**, *i.e., by*

19 $$\begin{aligned} \text {prob}\{\xi _{*}, t\} = \omega _{\,t}(\pi _{\xi _{*}}). \end{aligned}$$

$$\square $$

#### Remark

In *local relativistic quantum theory*, this axiom has to be replaced by a somewhat similar, though rather more subtle one, which incorporates the structure of the bundle of light cones in space-time and Einstein causality in a non-trivial way; see [13]. We will return to this topic in forthcoming work.

The *ETH*-Approach to QM yields the following picture of the *dynamics of states* in Quantum Mechanics: The evolution of states of an isolated open system *S* featuring actual events, in the sense of Definition 6 stated above, is determined by a (continuous-time) *stochastic branching process*, whose state space is referred to as the *non-commutative spectrum*, $\mathfrak {Z}_{S}$, of *S* (see [11]). Assuming that all the algebras $\mathcal {E}_{\ge t}$ are isomorphic to one specific (universal) von Neumann algebra, denoted by $\mathcal {N}$,^14^ the non-commutative spectrum, $\mathfrak {Z}_{S}$, of *S* is defined by

20 $$\begin{aligned} \mathfrak {Z}_{S}:= \bigcup _{\omega } \Big (\,\omega , \mathcal {Z}_{\omega }(\mathcal {N})\Big )\,, \end{aligned}$$

where the union over $\omega$ is a disjoint union, and $\omega$ ranges over *all* states of *S* of physical interest.^15^ Born’s Rule (19), together with Eqs. (12) and (16), then specifies the *branching probabilities* of the process. (See also Sects. 5 and 6.)

#### Remarks

1. Here is an explanation of the meaning of the name “*ETH*-Approach”: “*E*” stands for “events”, “*T*” for “trees”—referring to the tree-like structure of the space of all actualities an isolated physical system could in principle encounter in the course of its evolution—, and “*H*” stands for “histories”—referring to the *actual trajectory* of states occupied by the system in the course of its evolution.—The *ETH*-Approach represents a *completion* of Quantum Mechanics, which solves, for example, the “measurement problem,” rather than merely another “interpretation.”
2. **Axiom CP** (the State-Reduction-, or Collapse Postulate) formulated above, in combination with Eqs. (18) and (19), is reminiscent of the collapse postulate in the Copenhagen interpretation of QM. But, thanks to the Principle of Diminishing Potentialities (*PDP*), its status in the *ETH*-Approach to QM is not only logically consistent, but perfectly natural (i.e., not *ad hoc*). That the *non-commutative spectrum* $\mathfrak {Z}_S$ plays a very important role in an analysis of the time evolution of states becomes strikingly clear in local relativistic quantum theory; see [13].
  One might argue that the *ETH*-Approach to QM, in particular (*PDP*) and the Collapse Postulate, provides a mathematically precise version of the *Many-Worlds Interpretation* of QM, in that it specifies a precise rule for “branching.” However, in the *ETH*-Approach, there is no reason, whatsoever, to imagine that many alternative worlds actually *exist*!
3. In [11, 13], we have explained in which way the occurrence of an actual event may be detected through a (projective) measurement of a physical quantity. But, in general, there are plenty of actualities happening that are *not* related to the measurement of a previously specified physical quantity. Furthermore there is *no fundamental role* to be played by “observers” (let alone their consciousness) in the *ETH*-Approach to QM.
  An analysis of observations and measurements in QM and of how measurements are used to record events in the *ETH*-Approach has been presented in [11, 13] (see, in particular, Sect. 5 of [13]). It will not be repeated here. Suffice it to say that an *actuality* setting in at time *t*, described by a partition of unity $\big \{\pi _{\xi }\,\vert \, \xi \in \mathfrak {X}\big \}\subset \mathcal {E}_{\ge t}$, corresponds to measuring a physical quantitiy $\hat{X}\in \mathcal {O}_S$ (see Eq. (1)) iff the projections $\big \{\pi _{\xi }\,\vert \, \xi \in \mathfrak {X}\big \}$ can be well approximated (in the norm on the linear space $B(\mathcal {H}_S)$ given by the scalar product induced by the state $\omega _t$—see [11, 13]) by *spectral projections* of the self-adjoint operator $X(t')\in \mathcal {E}_{\ge t}$ representing $\hat{X}$ at some time $t'\gtrapprox t$. This will be clarified in Sect. 5 in the context of simple models.
4. We hope that the stochastic branching processes on the non-commutative spectra of isolated open systems derived from (*PDP*) and the Collapse Postulate, along with Eqs. (16), (17) and (19), will attract the interest of mathematicians. A beginning of an analysis of the simplest such processes is presented in Sect. 5.

## Huygens’ Principle and the Principle of Diminishing Potentialities

“*... principles are tested by inferences which are derivable from them. The nature of the subject permits of no other treatment.”* (Christiaan Huygens)

In this section, we explain why and how the existence of massless modes (photons or gravitons) in an isolated physical system implies the validity of the Principle of Diminishing Potentialities (*PDP*). We introduce a class of models of isolated systems for which this claim can be verified explicitly. The material discussed in this section also serves to motivate the models studied in Sect. 5.

We consider an isolated system, *S*, consisting of a very heavy (actually infinitely heavy) atom interacting with the quantized electromagnetic field. The atom is located in a compact region centered at the origin, $\mathbf {x}=\mathbf {0}$, of physical space $\mathbb {E}^{3}$. Gravitational effects are neglected. Points in space-time, $\mathbb {M}^{4}$, (Minkowski space) are denoted by $x=(x^{0}\equiv c\, t, \mathbf {x})$, where *c* is the speed of light, and $\mathbf {x}=(x^1,x^2,x^3)$ is a point in physical space $\mathbb {E}^{3}$. Let $F_{\mu \nu }(x) \equiv F_{\mu \nu }(\mathbf {x}, t)$ be the field tensor of the quantized free electromagnetic field, which is an operator-valued distribution. If $\big \{h^{\mu \nu }(\mathbf {x},t)\,\vert \,\mu , \nu = 0,1,2,3\big \}$ are real-valued test functions on $\mathbb {M}^{4}$ then

21 $$\begin{aligned} F(h):= \int _{\mathbb {M}^{4}} d^{4}x \,F_{\mu \nu }(\mathbf {x},t) \, h^{\mu \nu }(\mathbf {x},t) \end{aligned}$$

turns out to be a self-adjoint operator on the *Fock space*, $\mathcal {F}$, of the free electromagnetic field; (see, e.g., [34]). We may then consider bounded functions of the operators *F*(*h*), which are bounded operators on $\mathcal {F}$.

In a space-time description, the system *S* is located, at time *t*, in a compact region of $\mathbb {M}^{4}$ centered at $x=(c\, t, \mathbf {x}= \mathbf {0})$. Let $V^{+}_t$ be the (closure of the interior of the) forward light cone with vertex at the space-time point $(c\, t, \mathbf {0})$, and, likewise, let $V^{-}_t$ be the backward light cone with vertex at $(c\, t, \mathbf {0})$. For $t<t'$, we define the (space-time) *diamond* $D_{t,t'}$ by setting

22 $$\begin{aligned} D_{t,t'}:= V^{+}_t \cap V^{-}_{t'} \,. \end{aligned}$$

We define $\mathcal {A}_{[t,t']}$ to be the von Neumann algebra generated by all bounded functions of the operators *F*(*h*), where $\big \{h^{\mu \nu }(x)\,\vert \, \mu , \nu =0, 1, 2, 3, x \in \mathbb {M}^{4} \big \}$ are real-valued test functions on $\mathbb {M}^{4}$ with support in the diamond $D_{t, t'}$. For an arbitrary time $t\in \mathbb {R}$, we define the algebra $\mathcal {A}_{\ge t}$ to be the von Neumann algebra generated by all the algebras $\mathcal {A}_{[t,t']}, t'>t$; i.e.,

23 $$\begin{aligned} \mathcal {A}_{\ge t} := \overline{\bigvee _{\mathbb {R}\ni t'>t} \mathcal {A}_{[t,t']}}\,. \end{aligned}$$

We suppose that, besides the quantized electromagnetic field, *S* has “internal” degrees of freedom corresponding to excited states of the atom. Transitions between these states are described by operators acting on a (possibly only finite-dimensional) Hilbert space $\mathfrak {h}_S$. The Hilbert space of pure state vectors of *S* is thus given by

$$\begin{aligned} \mathcal {H}_S = \mathcal {F} \otimes \mathfrak {h}_S\,. \end{aligned}$$

Operators representing physical quantities characteristic of *S* generate algebras of operators acting on $\mathcal {H}_S$ that are defined as follows:

24 $$\begin{aligned} \mathcal {D}_{[t,t']}^{(0)}:=&\mathcal {A}_{[t,t']} \otimes \mathbf {1}\vert _{\mathfrak {h}_S}\,,\quad \mathcal {E}_{[t,t']}^{(0)} := \mathcal {A}_{[t,t']} \otimes B(\mathfrak {h}_S)\,,\quad \text {for arbitrary }\, t<t' ,\nonumber \\ \mathcal {E}_{\ge t}^{(0)}&:= \mathcal {A}_{\ge t} \otimes B(\mathfrak {h}_S), \quad \, \text {for all times } \, t , \quad \,\mathcal {E}^{(0)}\equiv \mathcal {E}:= B(\mathcal {H}_S)\,. \end{aligned}$$

The algebra $\mathcal {E}_{\ge t}^{(0)}$ may be interpreted as the *algebra of all potentialities at times* $\ge t$, as long as the internal degrees of freedom of the atom are not coupled to the electromagnetic field; (see Eqs. (10), (11), Sect. 3).

If $\mathfrak {M} \subset B(\mathcal {H}_S)$ is a (von Neumann) algebra of operators acting on $\mathcal {H}_S$ then $\mathfrak {M}\,'$ is defined to be the (von Neumann) algebra of *all* bounded operators on $\mathcal {H}_S$ commuting with *all* operators in $\mathfrak {M}$. The following lemma is a straightforward exercise.

### Lemma 2

(“Huygens’ Principle”). For arbitrary times *t* and $t'$, with $t' > t$,

25 $$\begin{aligned} \big [ \mathcal {E}_{\ge t'}^{(0)}\big ]' \cap \mathcal {E}_{\ge t}^{(0)} = \mathcal {D}_{[t,t']}^{(0)} \end{aligned}$$

### Sketch of proof:

Obviously, every operator in $\big [ \mathcal {E}_{\ge t'}^{(0)}\big ]'$ must commute with all operators of the form $\mathbf {1}\vert _{\mathcal {F}} \otimes C, \, C \in B(\mathcal {H}_S)$, i.e., it must have the form $A\otimes \mathbf {1}\vert _{\mathfrak {h}_S}$, where *A* is a bounded function of the electromagnetic field operators. If *A* belongs to $\mathcal {A}_{\ge t}$, as it must if $A\otimes \mathbf {1}$ belongs to $\mathcal {E}_{\ge t}^{(0)}$, then *A* is a bounded function of the field operators *F*(*h*), for test functions $\big \{h^{\mu \nu }\big \}$ supported in $V^{+}_{t}$. For the free electromagnetic field, a field operator *F*(*h*) commutes with *all* field operators *F*(*g*) affiliated^16^ with $\mathcal {A}_{\ge t'}$, i.e., with supp$(g^{\mu \nu }) \subseteq V^{+}_{t'}$, for $\mu , \nu = 0,1,2,3$, if and only if supp$(h^{\mu \nu }) \subseteq V^{-}_{t'}$, $\forall \, \mu , \nu$. This is a straightforward consequence of the fact that the commutator distributions (which, for the free electromagnetic field, are all proportional to a c-number distribution on $\mathbb {M}^{4}$ solving the wave equation) satisfy

26 $$\begin{aligned} \big [F_{\mu \nu }(x), F_{\rho \sigma }(y)\big ] = 0, \qquad \text {unless }\,\,(x-y)^{2} =0\,, \end{aligned}$$

i.e., unless $x-y$ is a lightlike vector. This is called *Huygens’ Principle* in quantum field theory. Thus, if *F*(*h*) is affiliated with $\mathcal {A}_{\ge t}$ and commutes with all operators in $\mathcal {A}_{\ge t'}$ then

27 $$\begin{aligned} \text {supp}(h^{\mu \nu }) \subseteq V^{+}_t \cap V^{-}_{t'} = D_{[t,t']}\,, \qquad \forall \, \mu , \nu \,. \end{aligned}$$

Bounded functions of the operators $F(h)\otimes \mathbf {1}\vert _{\mathfrak {h}_S}$, with $\big \{h^{\mu \nu }\big \}$ a real-valued test function satisfying (27), generate the algebra $\mathcal {D}_{[t,t']}^{(0)}$, and it follows from Eq. (26) and results in [35] that they commute with all operators in $\mathcal {E}_{\ge t'}^{(0)}$.

This completes our sketch of the proof of the lemma. $\square$

We note that the algebras $\mathcal {D}_{[t,t']}^{(0)}$ are infinite-dimensional.^17^ Lemma 2 and this last fact show that a very strong form of the *Principle of Diminishing Potentialities*,

$$\begin{aligned} \mathcal {E}_{\ge t'}^{(0)} \subsetneqq \mathcal {E}_{\ge t}^{(0)} \subsetneqq \mathcal {E}^{(0)}= B(\mathcal {H}_S), \quad \text {whenever }\,\,\, t< t' < \infty \,, \end{aligned}$$

holds for the system considered here, as long as the atom is not coupled to the quantized radiation field.

So far, only the free electromagnetic field has played a role in our discussion. The dynamics of the “internal” degrees of freedom of the system *S* described by operators acting on $\mathfrak {h}_S$ will be specified next, and we also describe how these degrees of freedom of *S* are coupled to the electromagnetic field. Let $H_0$ denote the usual Hamiltonian of the free electromagnetic field; ($H_0\ge 0$ is a self-adjoint operator on $\mathcal {H}_S$, see, e.g., [34]). We imagine that the internal degrees of freedom of the atom are driven in time in cycles of length *T*, which (w.l.o.g.) we may set to 1. We choose a unitary operator $U\equiv U_{1}\in \mathcal {E}_{[0,1]}^{(0)}$ and define

28 $$\begin{aligned} U_k:= e^{i(k-1)H_0}&U_{1} e^{-i(k-1)H_0}\,, \quad k=1,2,3, \dots \,, \quad U(n):=\prod _{k=1}^{n} U_{k} \,,\, \text { with }\, U(0):=\mathbf {1}\,,\nonumber \\ \Gamma :=&e^{-iH_0}U_1, \quad \Gamma ^{*}=\Gamma ^{-1}, \quad \Gamma ^{n} = e^{-inH_0} U(n)\,,\quad n=0,1, 2,3,\dots \,. \end{aligned}$$

Notice that

$$\begin{aligned} \Gamma ^{n}\cdot \Gamma ^{m}= \Gamma ^{n+m}\,, \qquad \forall \,\, n, m \quad \text {belonging to}\quad \mathbb {Z}. \end{aligned}$$

We consider $\big \{ \Gamma ^{n} \big \}_{n\in \mathbb {Z}}$ to be the propagator of *S*, and conjugation with this propagator describes the time evolution of operators in the Heisenberg picture when the atom is coupled to the electromagnetic field. At time $t=0$, *S* is prepared in a state $\omega _0$ given, for example, by

29 $$\begin{aligned} \omega _{0}(X):= \text {tr}(\big [\,\vert 0\rangle \langle 0\vert \otimes \widetilde{\Omega }\,\big ] \, X)\,, \end{aligned}$$

where *X* is an arbitrary operator in $\mathcal {E}_{\ge 0}:= \mathcal {E}_{\ge 0}^{(0)}$, $\vert 0\rangle$ is the vacuum vector in Fock space $\mathcal {F}$, and $\widetilde{\Omega }$ is some density matrix on $\mathfrak {h}_S$. To simplify our analysis, we henceforth regard time as discrete, $t=n \in \mathbb {Z}_{+}:=\{0,1,2, \dots \}$, and monitor the evolution of *S* only for non-negative times.

In accordance with the definition of the propagator $\big \{\Gamma ^{n}\big \}_{n\in \mathbb {Z}_{+}}$ in Eq. (28), we define algebras of potentialities, $\mathcal {E}_{\ge n}$, at times $\ge n$, with $n=0,1,2,\dots$, for the *interacting system* as follows:

30 $$\begin{aligned} \mathcal {E}:= \mathcal {E}_{\ge 0}^{(0)}\,, \quad \mathcal {E}_{\ge n} := \Big \{\Gamma ^{-n}\, X\, \Gamma ^{n}\, \vert \,X \in \mathcal {E} \Big \}\,, \quad \text {with }\,\, \Gamma ^{-1}= \Gamma ^{*}. \end{aligned}$$

Apparently, the algebras ${\mathcal {E}}_{\ge n}$ are related to the algebras $\mathcal {E}_{\ge n}^{(0)}$ by unitary conjugation, the unitary operator being *U*(*n*), i.e., every operator $Z\in {\mathcal {E}}_{\ge n}$ is of the form

$$\begin{aligned} Z= U(n)^{*} X U(n),\qquad \text {for some }\,\, X\in \mathcal {E}_{\ge n}^{(0)}\,, \end{aligned}$$

for arbitrary $n\in \mathbb {Z}_{+}$, as follows from (28). For arbitrary $n'>n\ge 0$, we define the algebra $\mathcal {D}_{[n,n']}$ to be given by conjugating all operators in $\mathcal {D}_{[n,n']}^{(0)}$ by the unitary operator $U(n')$. With these definitions, Lemma 2 implies that, for the interacting system, too, the Principle of Diminishing Potentialities, see Eq. (14), holds for all times *n* and $n'$, with $n'>n\ge 0$, and we have that

$$\begin{aligned} \big [\mathcal {E}_{\ge n'}\big ]' \cap \mathcal {E}_{\ge n} \simeq \mathcal {D}_{[n,n']}\,. \end{aligned}$$

It is important to note that

31 $$\begin{aligned} \Gamma ^{\,n-n'}\,\mathcal {E}_{\ge n}\, \Gamma ^{\,n'-n} = \mathcal {E}_{\ge n'} \subsetneqq \mathcal {E}_{\ge n}\,, \quad \text { for }\,\, n'>n\,. \end{aligned}$$

If time is restricted to the integers ($t=n\in \mathbb {Z}_+$) the prescriptions in Definition 6, Eqs. (16) and (17), and in **Axiom CP**, Eq. (19), of Sect. 3 determine a *stochastic branching process* on the non-commutative spectrum $\mathfrak {Z}_S:= \bigcup _{\omega } \big (\,\omega , \mathcal {Z}_{\omega } (\mathcal {N})\,\big )$, where $\mathcal {N} \simeq \mathcal {E}_{\ge 0}^{(0)}$, see (20), and $\omega$ ranges over all states given by density matrices on $\mathcal {H}_S$. It is quite subtle to describe this process explicitly, because it is *“non-Markovian”*, i.e., it has *memory.* To understand this claim we choose two intergers $\ell$ and *m*, with $\ell < m$, and consider the algebras

$$\begin{aligned} \mathcal {A}_{\le \ell } :=\overline{\bigvee _{\{k\vert k< \ell \}} \mathcal {A}_{[k,\ell ]}}\,, \quad \text {and }\,\, \mathcal {A}_{\ge m}\,, \end{aligned}$$

see Eq. (23). The arguments used in the proof of Lemma 2 show that

$$\begin{aligned} \mathcal {A}_{\le \ell } \subset \big (\mathcal {A}_{\ge m}\big )'\,, \end{aligned}$$

whenever $\ell < m$. A product state, $\varphi$, on $B(\mathcal {F})$ is a state with the property

32 $$\begin{aligned} \varphi (A \cdot B) = \varphi (A)\cdot \varphi (B)\,, \qquad \forall \, \,A \in \mathcal {A}_{\le \ell }, \, \,\, \forall \,\, B \in \mathcal {A}_{\ge m}\,,\quad \ell < m. \end{aligned}$$

It turns out that there are *no product states* (among all states of physical interest [23]); in particular, the state $\omega _0$ defined in (29) is *not* a product state. It is this fact that implies that, in the model considered here, there are memory effects in the stochastic evolution of states determined by the law encoded in Eqs. (16), (17) and (19) of Sect. 3. It should be stressed, furthermore, that this evolution is typically non-linear. *Only* for propagators $\big \{\Gamma ^{n}\big \}_{n\in \mathbb {Z}_+}$ that do *not* create any entanglement between the internal degrees of freedom of the atom and the electromagnetic field, the evolution of states of *S* in the *Schrödinger picture* is described by the usual unitary Schrödinger evolution.

We will not present a more detailed study of the models described above in the present paper.

In the next section we simplify matters by studying models that arise in the limit

33 $$\begin{aligned} c\, \rightarrow \, \infty \,, \end{aligned}$$

where *c* is the speed of light. In this limit, the diamonds $D_{t,t'}$ open up to entire *time slices*; i.e.,

$$\begin{aligned} D_{t,t'}= \big \{(\tau , \mathbf {x})\, \vert \, t \le \tau \le t', \mathbf {x} \in \mathbb {E}^{3}\big \}\,. \end{aligned}$$

If we require—as we will—that Lemma 2 continues to hold in the limit considered in (33) the algebras $\mathcal {D}_{[t,t']}$ must be contained in the commutant of all the algebras $\mathcal {D}_{[s,s']}$, whenever $t<t'\le s<s'$. It turns out that the resulting limiting models have plenty of *product states* of physical interest satisfying (32). The price to be payed is that the Hamiltonian, $H_{0}^{c=\infty }$, of the caricature of the radiation field corresponding to the limit (33) is *unbounded* above *and* below. (It has the same spectrum as the usual momentum operator.) This appears to be a general feature of models of non-relativistic QM satisfying the Principle of Diminishing Potentialities.

## Simple Models Illustrating the $\mathbf {\textit{ETH}}$-Approach to Quantum Mechanics

“*One of the characteristic traits of collapse models is radiation emission from any charged particle induced by the noise causing the collapse of the wave function.”* (Introduction to [8])

In this section we study a system *S* that is composed of a very heavy atom (as in Sect. 4) coupled to a caricature of the quantized electromagnetic field, hereafter called *“R-field”* (for “radiation field”), obtained in the limit (33) of the speed of light, *c*, tending to infinity. We introduce and analyze a class of simple models of *S* that supply examples of explicit dynamical laws governing the time evolution of states of *S* and hence illustrate the *ETH*-Approach to non-relativistic QM described in Sect. 3. In order to be able to carry out detailed calculations without appealing to high-brow mathematics, we adopt some rather drastic *simplifications*:

1. Time *t* is discrete: the time axis $\mathbb {R}$ is replaced by $\mathbb {Z}$.
2. The Hilbert space, $\mathfrak {h}_S$, of the internal degrees of freedom of the atom is finite-dimensional,
  34 $$\begin{aligned} \mathfrak {h}_S \simeq \mathbb {C}^{M}, \qquad \text {for some }\,\, M<\infty \,. \end{aligned}$$
  Physical quantities of the system *S* referring to the atom are described by self-adjoint operators on $\mathfrak {h}_S$, i.e., by hermitian $M\times M$ matrices. General states of the atom are described by density matrices, $\widetilde{\Omega }$, acting on $\mathfrak {h}_S$.
3. The algebra $\mathcal {A}_{[n, n+1]}$ generated by functions of the *R*-field localized in the time slice $[n,n+1]$ is chosen to be finite-dimensional, namely
  35 $$\begin{aligned} \mathcal {A}_{n}\equiv \mathcal {A}_{[n,n+1]} \simeq \mathbb {M}_{N}(\mathbb {C}), \qquad \text {for some }\,\, N<\infty \,, \end{aligned}$$
  i.e., by all $N\times N$ complex matrices acting on the *N*-dimensional Hilbert space $\mathcal {H}_n \simeq \mathbb {C}^{N}$.^18^ We choose an orthonormal basis $\big \{\phi _j\big \}_{j=0}^{N-1}$ in $\mathbb {C}^{N}$. The *“vacuum vector”* of the *R*-field is then defined by
  36 $$\begin{aligned} \vert 0 \rangle \equiv \Phi _{\underline{0}}:= \bigotimes _{n\in \mathbb {Z}} \phi _{k_n =0}. \end{aligned}$$
  The interpretation of the vacuum vector $\Phi _{\underline{0}}$ is that it is the state where no modes of the *R*-field are excited. Taking this vector as a so-called *reference vector*, the Hilbert space, $\mathfrak {F}_{S}$, of pure state vectors of the *R*-field is defined as follows: Let $\mathcal {S}_{fin}$ be the set of infinite sequences $\underline{k}:= \big \{k_n\big | k_n\in \{0, \dots , N-1\}\big \}_{n\in \mathbb {Z}}$ with the property that $k_n =0$, except for finitely many values of *n*. To a sequence $\underline{k}\in \mathcal {S}_{fin}$ we associate a (tensor-) product vector $\Phi _{\underline{k}}$ by setting
  37 $$\begin{aligned} \Phi _{\underline{k}}:= \bigotimes _{n\in \mathbb {Z}} \phi _{k_n}\,,\qquad \underline{k} \in \mathcal {S}_{fin}\,. \end{aligned}$$
  Every such vector belongs to the non-separable Hilbert space $\mathfrak {F}_{\infty }:=\bigotimes _{n\in \mathbb {Z}} \mathcal {H}_n$. Let $\mathfrak {D}$ denote the linear subspace of $\mathfrak {F}_{\infty }$ consisting of all *finite* linear combinations of vectors $\Phi _{\underline{k}}$, with $\underline{k}\in \mathcal {S}_{fin}$; $\mathfrak {D}$ is equipped with a scalar product $\langle \cdot , \cdot \rangle$ determined by
  38 $$\begin{aligned} \langle \Phi _{\underline{k}}, \Phi _{\underline{k}'}\rangle = \prod _{n \in \mathbb {Z}} \delta _{k_n, k'_n}\,, \end{aligned}$$
  which is extended to $\mathfrak {D}\times \mathfrak {D}$ anti-linearly in the first argument and linearly in the second argument. The Hilbert space $\mathfrak {F}_{S}$ is then defined to be the completion of the space $\mathfrak {D}$ in the norm given by
  $$\begin{aligned} \Vert \Psi \Vert := \sqrt{\langle \Psi , \Psi \rangle }, \qquad \Psi \in \mathfrak {D}\,. \end{aligned}$$
  The vectors $\big \{\Phi _{\underline{k}}\,\vert \, \underline{k}\in \mathcal {S}_{fin}\big \}$ form an orthonormal basis in $\mathfrak {F}_S$, hence $\mathfrak {F}_S$ is separable.

### Remark

It is interesting to consider other choices of reference vectors, $\Phi$, in the definition of the Hilbert space $\mathfrak {F}_S$. Examples will be given at the end of this section.

The total Hilbert space of the system *S* is defined by

39 $$\begin{aligned} \mathcal {H}_S:= \mathfrak {F}_{S}\otimes \mathfrak {h}_S\,. \end{aligned}$$

### Choice of algebras of operators representing potential events

*“With respect to the property of Direction, the ‘possible’ is called the Future and the ‘actualized’ the Past.”* (Oswald Spengler)

Next, we introduce algebras of operators appropriate to describe the system *S*, assuming first that the atom is *not* coupled to the *R*-field. Our definitions are similar to those introduced in Sect. 4.

#### Definition 7

*(Algebras generated by the* *R-field)*. For every integer *j*, we embed the algebra $\mathcal {A}_j\simeq \mathbb {M}_{N}(\mathbb {C})$ into the algebra $B(\mathfrak {F}_S)$ by taking the tensor product of any operator $F\vert _{\mathcal {H}_j} \in \mathcal {A}_j$ with the identity operators $\mathbf {1}\vert _{\mathcal {H}_{\ell }}$ on all spaces $\mathcal {H}_{\ell }$, with $\ell \not = j$. The resulting algebra is again denoted by $\mathcal {A}_j$. We then set

$$\begin{aligned} \mathcal {A}_{[n,n']}:= \bigotimes _{j=n}^{n'-1} \mathcal {A}_{j}\,, \quad \text {for }\,\, n'> n\,, \qquad \mathcal {A}_{\ge n}:= \overline{\bigvee _{n'>n} \mathcal {A}_{[n,n']}}\,, \end{aligned}$$

the closure being taken in the weak topology of $B(\mathfrak {F}_S)$. As in Eq. (24), we introduce the following algebras.

40 $$\begin{aligned} \mathcal {D}_{[n,n']}^{(0)}:= \mathcal {A}_{[n,n']} \otimes \mathbf {1}\vert _{\mathfrak {h}_{S}}&,\quad \mathcal {E}_{[n,n']}^{(0)}:= \mathcal {A}_{[n,n']}\otimes B(\mathfrak {h}_{S}), \quad n<n'\,,\nonumber \\&\mathcal {E}_{\ge n}^{(0)}:=\mathcal {A}_{\ge n} \otimes B(\mathfrak {h}_S)\,. \end{aligned}$$

$$\square $$

One immediately checks that

41 $$\begin{aligned} \big [\mathcal {E}_{\ge n'}^{(0)}\big ]' \cap \mathcal {E}_{\ge n}^{(0)} = \mathcal {D}_{[n,n']}^{(0)}, \qquad \forall n'>n\,. \end{aligned}$$

This imples that the *Principle of Diminishing Potentialities (PDP)*—see Eq. (14) of Sect. 3—holds in this model, as long as the atom is not coupled to the *R*-field, yet.

We observe that the algebras $\mathcal {A}_{[n,n']}$ are isomorphic to $B(\bigotimes _{j=n}^{n'-1}\mathcal {H}_{j})$, for arbitrary $n<n'$, and that the states

42 $$\begin{aligned} \varphi _{\underline{k}}(F):= \langle \Phi _{\underline{k}}, F\, \Phi _{\underline{k}} \rangle \,, \,\, \qquad F \in B(\mathfrak {F}_S)\,, \end{aligned}$$

are *product states*, in the sense of Eq. (32), Sect. 4. For these reasons, there are no memory effects in the time evolution of states of the *R*-field, taken to be density matrices on $\mathfrak {F}_S$, asymptotically as time *t* tends to $\infty$. This will be used in Subsect. 5.3.

In the following, we will only monitor the time evolution of states of *S* for times $t\ge t_{in}$, where $t_{in}$ is an *initial time* that, in the following, we set to 0, and we then choose the algebra of physical quantities characteristic of *S* to be given by $\mathcal {E}= \mathcal {E}_{\ge 0}^{(0)}$.

### Time evolution in the Heisenberg picture

*“The constant element in physics, since Newton, is not a configuration or a geometrical form, but a law of dynamics.”* (Werner Heisenberg)

Next, we describe the Heisenberg-picture time evolution of operators representing physical quantities characteristic of the system *S*; (see Eq. (4), Sect. 2). The “free” time evolution of the *R*-field, before it is coupled to the atom, is given in terms of a “shift operator,” $\mathfrak {S}$, on $\mathfrak {F}_S$:

43 $$\begin{aligned} \big (\mathfrak {S}\Phi \big )_{\underline{k}}:= \Phi _{\sigma (\underline{k})} = \bigotimes _{n\in \mathbb {Z}} \phi _{\sigma (\underline{k})_{n}}, \quad \text {with }\,\,\, \sigma (\underline{k})_n := k_{n+1}\,, \,\forall \, n\in \mathbb {Z}. \end{aligned}$$

The definition of the operator $\mathfrak {S}$ is extended to the domain $\mathfrak {D}$ (dense in $\mathfrak {F}_S$) by linearity. It is obviously unitary on $\mathfrak {D}$ and hence extends to all of $\mathfrak {F}_S$. We observe that $\mathfrak {S}$ leaves the vacuum (reference) vector $\vert 0 \rangle \equiv \Phi _{\underline{0}} \in \mathfrak {F}_S$ (see Eq. (36)) invariant. The shift operator $\mathfrak {S}$ is the analogue of the operator $e^{-iH_0}$ considered in Sect. 4. If time were chosen to be continuous then the generator of time evolution of the *R*-field would be an operator unitarily equivalent to a standard *momentum operator* and hence would be unbounded from above and from below.

For an arbitrary operator $F\in \mathcal {A}_{[n,n']}$, we set

$$\begin{aligned} F(t):=\big [\mathfrak {S}^{*}\big ]^{t}\,F\, \mathfrak {S}^{t}, \qquad t \in \mathbb {Z}\,, \end{aligned}$$

and find that $F(t) \in \mathcal {A}_{[n+t,n'+t]}$, for arbitrary $n<n'$. Let *V* be a unitary matrix on the atomic Hilbert space $\mathfrak {h}_S = \mathbb {C}^{M}$ describing the propagator of the atom by one time step. Before the atom is coupled to the *R*-field the Heisenberg-picture time evolution of bounded operators, *C*, on $\mathcal {H}_S$ is given by conjugation with the unitary propagator $\big \{\Gamma _{0}^{\,t}\big \}_{t\in \mathbb {Z}}$ on $\mathcal {H}_S$,

$$\begin{aligned} C(t):=\big [\Gamma _{0}^{*}\big ]^{t}\, C \, \Gamma _{0}^{\,t} = \Gamma _{0}^{-t}\,C\,\Gamma _{0}^{t}\,, \qquad t\in \mathbb {Z}\,, \end{aligned}$$

where

44 $$\begin{aligned} \Gamma _{0} := \mathfrak {S} \otimes V \end{aligned}$$

is a unitary operator on $\mathcal {H}_S$. We observe that

$$\begin{aligned} \mathcal {E}_{\ge (n+t)}^{(0)}=\Big \{\Gamma _{0}^{-t}\, X\, \Gamma _{0}^{\,t} \, \vert \,X \in \mathcal {E}_{\ge n}^{(0)}\Big \} ,\qquad \forall \, n, t \text { in }\, \mathbb {Z}\,. \end{aligned}$$

For $t\ge 0,\, \mathcal {E}_{\ge (n+t)}^{(0)}$ is contained in $\mathcal {E}_{\ge n}^{(0)}$ , i.e., Heisenberg-picture time evolution by a time step $t\ge 0$ of operators on $\mathcal {H}_S$ defines a $^{*}$*endomorphism* of $\mathcal {E}_{\ge n}^{(0)}$, for arbitrary $n \in \mathbb {Z}$. For a strictly positive *t*, $\mathcal {E}_{\ge (n+t)}^{(0)}$ is properly contained in $\mathcal {E}_{\ge n}^{(0)}$, and (*PDP*) holds.

Next, we introduce **interactions** between the atom and the *R*-field. As announced, we only monitor the evolution of states of *S* for non-negative times, $t\in \mathbb {Z}_{+}$. We choose a *unitary* operator *U* in the algebra $B(\mathbb {C}^{N}) \otimes B(\mathfrak {h}_S)$ and define $U_1$ to be the corresponding operator in the algebra $\mathcal {A}_{0} \otimes B(\mathfrak {h}_S)$; see Eq. (35). We define

45 $$\begin{aligned} U_k:= \Gamma _{0}^{1-k} \,U_1\, \Gamma _{0}^{k-1}, \,\,\, k=1,&2,3,\dots , \quad U(n):= U_{n} \cdots U_{1} \,, \,\,n=1,2, 3,\dots ,\nonumber \\&\Gamma := \Gamma _{0} U_1,\,\,\, \Gamma ^{-1}= \Gamma ^{*}\,, \end{aligned}$$

see Eq. (28) for comparison. Notice that

$$\begin{aligned} U_k \in \mathcal {A}_{k-1}\otimes B(\mathfrak {h}_S)\,, \quad k=1,2,3,\dots \end{aligned}$$

It is straightforward to verify that the operators $U(n), n=1,2, \dots ,$ and $\Gamma$ are unitary, and that

46 $$\begin{aligned} \Gamma ^{\,n}=\Gamma _{0}^{\,n} U(n)\,,\,\,\, \Gamma ^{-n}=(\Gamma ^{*})^{n},\,\,\,\forall \,\,\, n \in \mathbb {Z}_{+}\,. \end{aligned}$$

We interpret $\big \{\Gamma ^{\,n}\big \}_{n\in \mathbb {Z}}$ (with $\Gamma ^{0}:= \mathbf {1}$) as the unitary propagator of the system *S* in the presence of interactions between the idealized atom and the *R*-field.

#### Definition 8

Event algebras $\mathcal {E}_{\ge n}$ (with $n\ge 0$ henceforth) of the *interacting* system are defined by setting

47 $$\begin{aligned} \mathcal {E}:= \mathcal {E}_{\ge 0}^{(0)}\,, \quad \mathcal {E}_{\ge n} := \Big \{\Gamma ^{-n}\, X\, \Gamma ^{\,n}\, \vert \, X \in \mathcal {E} \Big \}\,,\quad n=0,1,2,\dots \,, \,\, \text { with } \,\,\,\, \Gamma ^{-1}= \Gamma ^{*}\,.\nonumber \\ \end{aligned}$$

$$\square $$

See also Sect. 4, Eq. (30).

From now on, we identify physical quantities, $\hat{X}$, characteristic of the system *S* with operators $X\equiv X(0) \in \mathcal {E}$ given by a sum of operators of the form $F\otimes C$, where $F\in \mathcal {A}_{\ge 0}$ and *C* is an $M\times M$ matrix on $\mathfrak {h}_S$; and we set $X(t)= \Gamma ^{-t}\,X\,\Gamma ^{\,t}\,, t\in \mathbb {Z}.$ We then have that

$$\begin{aligned} \mathcal {E}_{\ge n'} = \Gamma ^{-t}\, \mathcal {E}_{\ge n} \, \Gamma ^{\,t} \subseteq \mathcal {E}_{\ge n}\,, \quad \text {for }\,\, t= n'-n\ge 0\,, \end{aligned}$$

i.e., time evolution by a time step $t\ge 0$ is given by a $^{*}$endomorphism from $\mathcal {E}_{\ge n}$ to $\mathcal {E}_{\ge (n+t)}\subseteq \mathcal {E}_{\ge n}$.

We observe that every operator $Z\in \mathcal {E}_{\ge n}$ is of the form

48 $$\begin{aligned} Z= U(n)^{*}\, Y \,U(n),\quad \text {for some }\,\, Y\in \mathcal {E}_{\ge n}^{(0)}\,, \end{aligned}$$

see Eq. (46), and every $Y\in \mathcal {E}_{\ge n}^{(0)}$ can be written as

49 $$\begin{aligned} Y= \Gamma _{0}^{-n}\,X\,\Gamma _{0}^{n}, \qquad \text { for some }\, X\in \mathcal {E}\,, \end{aligned}$$

for arbitrary $n\in \mathbb {Z}_{+}$. We define

50 $$\begin{aligned} \mathcal {D}_{[n,n']}:= \big \{U(n')^{*} X U(n')\,\vert \, X \in \mathcal {D}_{[n,n']}^{(0)}\big \} \subset \mathcal {E}_{\ge n}\,, \quad 0\le n<n'\,. \end{aligned}$$

Equations (41), (47), (48) and (50) then imply that

$$\begin{aligned} \big [\mathcal {E}_{\ge n'}\big ]' \cap \mathcal {E}_{\ge n} = \mathcal {D}_{[n,n']}\,, \end{aligned}$$

and hence the *Principle of Diminishing Potentialities* holds in the model of the interacting system.

### The law of evolution of states according to the *ETH* approach

*“The idea that elimination of coherence, in one way or another, implies the replacement of ‘and’ by ‘or’, is a very common one among solvers of the ‘measurement problem’. It has always puzzled me.”* (John Stewart Bell)

In this and the following subsection, it is convenient to be able to also work in the *Schrödinger picture*, rather than only in the *Heisenberg picture* used so far.

*Remark on the Schrödinger picture.* General states of *S* are described by density matrices, $\widetilde{\Sigma }$, on $\mathcal {H}_S$. By $\widetilde{\Sigma }_{n}$ we denote the density matrix obtained by restricting the state corresponding to $\widetilde{\Sigma }$ to the algebra $\mathcal {E}_{\ge n}$. By $\Sigma _{n}$ we denote the *same* state of *S*, but viewed as a state in the *Schrödinger picture*, which is related to the *Heisenberg picture* by the following identity:

51 $$\begin{aligned} \text {tr}_{\mathcal {H}_S}\big (\widetilde{\Sigma }_{n}\cdot X(t)\big ) = \text {tr}_{\mathcal {H}_S} \big (\Sigma _{n}\cdot \big [\Gamma ^{*}\big ]^{t-n} \,X\, \Gamma ^{t-n}\big ) = \text {tr}_{\mathcal {H}_S} \big (\Gamma ^{t-n}\,\Sigma _{n} \, \big [\Gamma ^{*}\big ]^{t-n}\cdot X\big )\,, \quad t\ge n\,, \end{aligned}$$

for an arbitrary operator $X \in \mathcal {E}$.

In Eq. (51), $X(t)= \Gamma ^{-t} \,X\, \Gamma ^{t}$, $\Sigma _n := \Gamma ^{n}\, \Sigma _{0}\, \Gamma ^{-n}$, where $\Sigma _{0}\equiv \Sigma = \widetilde{\Sigma }\,,$ and we recall that $\mathcal {E}_{\ge n}=\big \{\Gamma ^{-n}\,X\,\Gamma ^{n}\,\big |\, X\in \mathcal {E}\big \}$.

Next, we describe the *time evolution of states*, as predicted by the *ETH*-Approach to QM summarized in Sect. 3. More specifically, using Definition 6 of actual events in Subsect. 3.2 and the Collapse Postulate, **Axiom CP**, in Subsect. 3.3, we will construct a trajectory, $\big \{\omega _n\, \big | \, n\in \mathbb {Z}_{+}\big \}$, of states, where $\omega _n$ is a state on the algebra $\mathcal {E}_{\ge n}$, with initial condition $\omega _{n=0}=\omega$, and $\omega$ is the state on the algebra $\mathcal {E}$ given by

52 $$\begin{aligned} \omega (X):= \text {tr}_{\mathcal {H}_S}\big ((P_{\underline{k}} \otimes \widetilde{\Omega })\cdot X\big ), \qquad X\in \mathcal {E}\,. \end{aligned}$$

In this equation, $P_{\underline{k}}$ denotes the orthogonal projection onto the vector $\Phi _{\underline{k}} \in \mathfrak {F}_S,\, \underline{k}\in \mathcal {S}_{fin}$, and $\widetilde{\Omega }\equiv \Omega$ is some density matrix on the Hilbert space $\mathfrak {h}_S$ of the atom. We remark that when evaluated on $\mathcal {E}= \mathcal {E}_{\ge 0}^{(0)}$ (see Eq. (40)) the state $\omega$ is *independent* of $\{k_n\}_{n<0}$.

We start our analysis by studying the restriction of the state $\omega$ defined in Eq. (52) to the algebra $\mathcal {E}_{\ge n}$, for some $n>0$. For this purpose, we introduce operators ($M\times M$ matrices), $\mathfrak {L}_{\alpha }^{\ell }$, on $\mathfrak {h}_S \simeq \mathbb {C}^{M}$ by specifying their matrix elements

53 $$\begin{aligned} \langle u, \mathfrak {L}_{\alpha }^{\ell } v \rangle := \langle \phi _{\alpha } \otimes u, U \,\phi _{\ell } \otimes v \rangle , \quad \alpha , \ell = 0,1,\dots , N-1, \,\, \,\text { for arbitrary }\,\, u, v \text { in }\,\,\mathfrak {h}_S\,,\nonumber \\ \end{aligned}$$

where *U* is the unitary operator describing the interaction between the atom and the *R*-field chosen right above Eq. (45). From expression (53) for $\mathfrak {L}_{\alpha }^{\ell }$, the unitarity of the operator *U* and the completeness relation $\sum _{\alpha =0}^{N-1} \vert \phi _{\alpha }\rangle \, \langle \phi _{\alpha }\vert = \mathbf {1}$ we infer that

54 $$\begin{aligned} \sum _{\alpha =0}^{N-1} [\mathfrak {L}_{\alpha }^{\ell }]^{*} \cdot \mathfrak {L}_{\alpha }^{\ell } = \mathbf {1}\vert _{\mathbb {C}^{M}}\,. \end{aligned}$$

The operators $\big \{\mathfrak {L}_{\alpha }^{\ell }\big \}_{\alpha =1}^{N}$ are called *Kraus operators* [36, 37]. Let $Z\in \mathcal {E}_{\ge n}$, then $\exists \, X\in \mathcal {E}$ such that

$$\begin{aligned} Z = \Gamma ^{-n}\, X\,\Gamma ^{\,n}, \end{aligned}$$

with $\Gamma$ as defined in Eq. (45). Any operator $X\in \mathcal {E}=\mathcal {E}_{\ge 0}^{(0)}= \mathcal {A}_{\ge 0}\otimes B(\mathfrak {h}_S)$ is a linear combination of operators of the form $F \otimes C$, where $F\in \mathcal {A}_{\ge 0}$ and *C* is an $M\times M$ matrix acting on $\mathfrak {h}_S$.

We first determine the restriction of the state $\omega$ to the algebra $\mathcal {E}_{\ge 1}$, (i.e., we set $n=1$). We choose an operator $Z:=\Gamma ^{-1} (F\otimes C)\Gamma$, with *F* and *C* as above. The density matrix $\widetilde{\Omega }$ can be diagonalized,

$$\begin{aligned} \widetilde{\Omega }= \sum _{j=1}^{M} p_j \vert v_j\rangle \langle v_j\vert , \qquad p_j \ge 0, \forall \, j, \quad \sum _{j=1}^{M}p_j =1\,, \end{aligned}$$

where $\big \{v_j \big \}_{j=1}^{M}$ is an orthonormal basis of eigenstates of $\widetilde{\Omega }$. By inserting the partition of unity $\sum _{\alpha =1}^{N} \vert \phi _{\alpha } \rangle \langle \phi _{\alpha }\vert = \mathbf {1}\vert _{\mathbb {C}^{N}}$ and using definition (53) of the operators $\mathfrak {L}_{\alpha }^{\ell }$ we show that

55 $$\begin{aligned} \text {tr}_{\mathcal {H}_S}\big (&(P_{\underline{k}}\otimes \widetilde{\Omega })\cdot \Gamma ^{-1}(F\otimes C)\Gamma \big )\nonumber \\&= \langle \Phi _{\underline{k}}, \mathfrak {S}^{-1} F\, \mathfrak {S} \Phi _{\underline{k}}\rangle \cdot \Big \{\sum _{j=1}^{M} p_{j}\, \langle (\mathbf {1}\otimes V)U(\phi _{k_0}\otimes v_j), (\mathbf {1}\otimes C) (\mathbf {1}\otimes V) U(\phi _{k_0} \otimes v_j )\rangle \, \nonumber \\&=\langle \Phi _{\sigma (\underline{k})}, F\, \Phi _{\sigma (\underline{k})}\rangle \cdot \Big \{\sum _{j=1}^{M} p_{j} \sum _{\alpha =1}^{N} \langle V\mathfrak {L}_{\alpha }^{k_0} v_j, C\, V\mathfrak {L}_{\alpha }^{k_0} v_j \rangle \Big \} \nonumber \\&= \langle \Phi _{\sigma (\underline{k})}, F\, \Phi _{\sigma (\underline{k})}\rangle \cdot \Big \{\sum _{\alpha =1}^{N} \text {tr}_{\mathfrak {h}_S} \big (V\mathfrak {L}_{\alpha }^{k_0}\, \Omega \, [V\mathfrak {L}_{\alpha }^{k_0} ]^{*} \cdot C\big )\Big \}\,, \end{aligned}$$

where $\Omega = \widetilde{\Omega }$ (at time $t=0$), and where *V* is the *time*-1 propagator of the atom decoupled from the R-field.

This calculation easily generalizes to arbitrary times $n>1$. Choosing $Z:= \Gamma ^{-n} (F\otimes C) \Gamma ^{n}$, with $F\in \mathcal {A}_{\ge 0}$ and $C\in B(\mathfrak {h}_{S})$, we find that

56 $$\begin{aligned} \omega (Z)=&\langle \Phi _{\underline{k}}, [\mathfrak {S}]^{-n} F\, \mathfrak {S}^{n} \Phi _{\underline{k}}\rangle \cdot \Big \{ \sum _{\alpha _1, \dots , \alpha _n} \text {tr}_{\mathfrak {h}_S} \Big (\widetilde{\Omega }\, \Big \{\prod _{\ell = 1}^{n} [V\,\mathfrak {L}_{\alpha _{\ell }}^{k_{\ell }}]^{*}\Big \}\, C\, \Big \{ \prod _{j=n}^{1} (V\, \mathfrak {L}_{\alpha _j}^{k_j})\Big \} \Big ) \Big \}\nonumber \\ =&\langle \Phi _{\sigma ^{n}(\underline{k})}, F \,\Phi _{\sigma ^{n}(\underline{k})} \rangle \cdot \Big \{\sum _{\alpha _1, \dots , \alpha _n} \text {tr}_{\mathfrak {h}_S} \Big (\Big \{V\,\mathfrak {L}_{\alpha _n}^{k_n} \cdots V\,\mathfrak {L}_{\alpha _1}^{k_1} \,\Omega \, [\mathfrak {L}_{\alpha _1}^{k_1}]^{*}V^{*}\cdots [\mathfrak {L}_{\alpha _n}^{k_n}]^{*}V^{*}\Big \}\cdot C\Big ) \Big \}\,, \end{aligned}$$

where the sum over $\alpha _j$ ranges over $\alpha _j =0,\dots , N-1,\,\, \forall \, j=1,\dots , n$. Formula (56) shows that the evolution of states is entangling the state of the R-field with the state of the atom, as one would expect when interactions between the *R*-field and the atom are turned on. Because of our special choice of the state of the *R*-field, $\Phi _{\underline{k}}$, which is a *(tensor) product state*—and *only* because of this feature—it is given by a *quantum Markov chain*. To determine the evolution of states predicted by the *ETH*-Approach we will have to “unravel” the evolution described by formula (56); see Theorem 4 below.

#### Lemma 3

The maps

57 $$\begin{aligned} \Omega \,\, \mapsto V\,\Omega \,V^{*}\quad \text { and }\quad \Omega \,\, \mapsto \sum _{\alpha } \mathfrak {L}_{\alpha }^{\ell }\,\Omega \, [\mathfrak {L}_{\alpha }^{\ell }]^{*} \end{aligned}$$

are completely positive and trace-preserving, so that the right sides in (57) are again density matrices on $\mathfrak {h}_S$. $\square$

This lemma has been established by *Kraus* in [36, 37]. It implies that the map

$$\begin{aligned} \Omega \,\,\mapsto \sum _{\alpha _1, \dots , \, \alpha _n} V\,\mathfrak {L}_{\alpha _n}^{k_n} \cdots V\,\mathfrak {L}_{\alpha _1}^{k_1} \,\Omega \, [\mathfrak {L}_{\alpha _1}^{k_1}]^{*}V^{*}\cdots [\mathfrak {L}_{\alpha _n}^{k_n}]^{*}V^{*} \end{aligned}$$

is completely positive and gives rise to a *quantum Markov chain*.

We remark, in passing, that the dynamics considered in Sect. 4 is *not* Markovian, which means that it is considerably more complicated to analyze it. The same remark applies if the state of the *R*-field is *not* a product state but entangles modes of the *R*-field localized in different time slices.

Next, we determine those states $\omega _n, n=0,1,2, \dots ,$ on the algebras $\mathcal {E}_{\ge n}$ that can be reached *recursively* from the initial condition $\omega _0 = \omega$, with $\omega$ as in Eq. (52), by applying the *law of evolution of states* of the *ETH*-Approach, as formulated in Definition 6 (actual events) and **Axiom CP** (Collapse Postulate) of Sect. 3. We use induction in time to accomplish this task, explaining the induction step from time $m-1$ to time *m* by outlining the construction of $\omega _m$, given $\omega _{m-1}$.

#### Theorem 4

Let $Z:= \Gamma ^{-n}(F\otimes C)\,\Gamma ^{n} \in \mathcal {E}_{\ge n}$, with $F\in \mathcal {A}_{\ge 0}$ and $C\in B(\mathfrak {h}_S)$ (a general element of $\mathcal {E}_{\ge n}$ being a sum of such operators), and let $\omega _0 =\omega$ be the state on the algebra $\mathcal {E}$ specified in Eq. (52). Let $\omega _n$ be a state obtained from $\omega _0$ by applying the law of evolution of states of the *ETH*-Approach formulated in Sect. 3. Then

58 $$\begin{aligned} \omega _{n}(Z)= \langle \Phi _{\sigma ^{n}(\underline{k})}, F \,\Phi _{\sigma ^{n}(\underline{k})}\rangle \cdot \text {tr}_{\mathfrak {h}_S}\big (\Omega _n \cdot C\big )\,, \end{aligned}$$

where $\Omega _{n} = \big [\text {tr}_{\mathfrak {h}_S} (\Pi ^{(n)})\big ]^{-1} \,\Pi ^{(n)}$, and $\Pi ^{(n)}$ is an orthogonal projection on the Hilbert space $\mathfrak {h}_S$ of the atom.

#### Proof

Theorem 4 is proven by induction in time $n\in \mathbb {Z}_{+}$. Equation (58) is our *induction hypothesis*, denoted $(\mathcal {I}_{n})$. Clearly $(\mathcal {I}_{n})$ holds for $n=0$. We assume that $(\mathcal {I}_{n})$ holds for $n=m-1$, for some $m=1,2,\dots$, and show that this implies that it holds for $n=m$. This is done in two steps: We first restrict the state $\omega _{m-1}$ to the algebra $\mathcal {E}_{\ge m}\subsetneqq \mathcal {E}_{\ge m-1}$, the resulting state on $\mathcal {E}_{\ge m}$ being denoted by $\widehat{\omega }_m$. We then apply **Axiom CP** (the Collapse Postulate) of Subsect. 3.3 to select a state $\omega _m$ subordinate to $\widehat{\omega }_m$ (written as $\omega _m \prec \widehat{\omega }_m$).

We now repeat steps very similar to those leading to Eq. (55) in more detail. From Eq. (48) we infer that an operator $\tilde{Z} \in \mathcal {E}_{\ge m}\subset \mathcal {E}_{\ge m-1}$ is a sum of operators of the form

59 $$\begin{aligned} \tilde{Z} = U(m-1)^{*} \,\Gamma _{0}^{1-m}\, \tilde{X}\,\Gamma _{0}^{m-1} U(m-1)\,, \end{aligned}$$

where

$$\begin{aligned} \tilde{X}:= U_{1}^{*}\, \big [(\mathfrak {S}^{*}F\mathfrak {S})\otimes V^{*}C\,V\big ]\,U_1 \,\text { belongs to }\, \mathcal {E}_{\ge 1}\subset \mathcal {E}, \end{aligned}$$

Thus, we can apply the induction hypothesis to $\omega _{m-1}(\tilde{Z})$. The density matrix $\Omega _{m-1}$ can be written as

60 $$\begin{aligned} \Omega _{m-1}= \sum _{j=1}^{M} p_j \vert v_j^{(m-1)}\rangle \langle v_j^{(m-1)} \vert \,, \end{aligned}$$

where $\big \{ v_{j}^{(m-1)} \big \}_{j=1}^{M}$ is a complete orthonormal system of eigenstates of $\Omega _{m-1}$, $p_j \ge 0,\,\forall \,j=1,\dots , M,$ with $\sum _{j=1}^{M}p_j =1$. Note that the induction hypothesis $(\mathcal {I}_{m-1})$ is linear in $\Omega _{m-1}$; see Eq. (58). Thus, for *Z* as in the statement of Theorem 4, $(\mathcal {I}_{m-1})$ can be written as

61 $$\begin{aligned} \omega _{m-1}(Z)&=\sum _{j=1}^{M} p_j \,\omega _{m-1}^{j}(Z)\,, \qquad \text {where} \nonumber \\ \omega _{m-1}^{j}(Z)&:= \langle \Phi _{\sigma ^{m-1}(\underline{k})}, F\,\, \Phi _{\sigma ^{m-1}(\underline{k})} \rangle \cdot \langle v_{j}^{(m-1)}, C\,v_{j}^{(m-1)}\rangle \,. \end{aligned}$$

If we now set $Z:=\tilde{Z}$, with $\tilde{Z}$ as specified in (59), the $j^{th}$ term on the right side is given by

62 $$\begin{aligned} \omega _{m-1}^{j}(\tilde{Z})&=\langle \Phi _{\sigma ^{m}(\underline{k})}, F\,\, \Phi _{\sigma ^{m}(\underline{k})} \rangle \cdot \langle U\,[ \phi _{\sigma ^{m-1}(\underline{k})_{0}}\otimes \,v_{j}^{(m-1)} ], \nonumber \\&\quad \big (\mathbf {1}\otimes V^{*}\,C\cdot V \big )\,U\,[\phi _{\sigma ^{m-1}(\underline{k})_{0}}\otimes \,v_{j}^{(m-1)} ]\rangle \end{aligned}$$

We recall that $\sigma ^{m-1}(\underline{k})_0 = k_{m-1}$. Next, we recall the definition of the Kraus operators $\mathfrak {L}_{\alpha }^{\ell }$ (see Eq. (53)) and use the completeness of $\big \{\phi _{\alpha }\big \}_{\alpha =0}^{N-1}$ to show that

$$\begin{aligned} \omega _{m-1}^{j}(\tilde{Z})= \langle \Phi _{\sigma ^{m}(\underline{k})}, F\,\, \Phi _{\sigma ^{m}(\underline{k})} \rangle \cdot \Big \{ \sum _{\alpha =0}^{N-1} \langle V\,\mathfrak {L}_{\alpha }^{{k}_{m-1}}\, v_{j}^{(m-1)}, C\,V\,\mathfrak {L}_{\alpha }^{{k}_{m-1}}\, v_{j}^{(m-1)}\rangle \Big \}\,. \end{aligned}$$

Defining

63 $$\begin{aligned} \widehat{\Omega }_{m}:= \sum _{\alpha =0}^{N-1} V\cdot \mathfrak {L}_{\alpha }^{k_{m-1}} \, \Omega _{m-1} \big (\mathfrak {L}_{\alpha }^{k_{m-1}}\big )^{*}\cdot V^{*}\,, \end{aligned}$$

and recalling (60) we conclude that

64 $$\begin{aligned} \widehat{\omega }_{m}(\tilde{Z})\equiv \omega _{m-1}(\tilde{Z})= \langle \Phi _{\sigma ^{m}(\underline{k})}, F\,\, \Phi _{\sigma ^{m}(\underline{k})} \rangle \cdot \text {tr}_{\mathfrak {h}_S} \big (\widehat{\Omega }_{m} \cdot C\big )\,. \end{aligned}$$

Lemma 3 tells us that the right side of (63) defines a density matrix on $\mathfrak {h}_S$. Let

65 $$\begin{aligned} \widehat{\Omega }_{m} = \sum _{r=1}^{s} q_{r}^{(m)} \Pi _{r}^{(m)}, \qquad s\le M\,, \end{aligned}$$

be the spectral decomposition of $\widehat{\Omega }_m$, with $\Pi _{r}^{(m)}$ the spectral projection of $\widehat{\Omega }_m$ corresponding to the eigenvalue $q_{r}^{(m)}$. The eigenvalues of $\widehat{\Omega }_{m}$ are ordered such that

$$\begin{aligned} q_{1}^{(m)}> q_{2}^{(m)}> \dots q_{s}^{(m)} > 0,\quad \text {and we have that }\quad \sum _{r=1}^{s} q_{r}^{(m)} \,\text {tr}_{\mathfrak {h}_S}\big (\Pi _{r}^{(m)}\big ) =1. \end{aligned}$$

We set $\Pi _{0}^{(m)}:= \mathbf {1}- \sum _{r=1}^{s} \Pi _{r}^{(m)}$. Let $P_{\underline{k}}$ be the rank-1 orthogonal projection onto $\Phi _{\underline{k}}$ and $P_{\underline{k}}^{\perp }: =\mathbf {1}- P_{\underline{k}}$. Using Definition 6 (actual events) of Sect. 3, we find that, in the *Schrödinger picture*, the actual event happening at time *m* is described by the family of orthogonal projections

66 $$\begin{aligned} \big \{P_{\sigma ^{m}(\underline{k})} \Pi _{r}^{(m)}, P_{\sigma ^{m}(\underline{k})}^{\perp } \Pi _{r}^{(m)}\big \}_{r=0}^{s}\,, \end{aligned}$$

which generate an algebra unitarily conjugated to the center, $\mathcal {Z}_{\widehat{\omega }_m}(\mathcal {E}_{\ge m})$, of the centralizer of $\widehat{\omega }_m$, with $\widehat{\omega }_m :=\omega _{m-1}\vert _{\mathcal {E}_{\ge m}}$. We now apply **Axiom CP** (the Collapse Postulate) formulated in Subsect. 4.3: The probability of the state $\widehat{\omega }_m$ collapsing onto the range of a projection proportional to $P_{\sigma ^{m}(\underline{k})}^{\perp }$ or to $\Pi _{0}^{(m)}$ vanishes, as follows from (64) and *Born’s Rule* (see Eq. (19), Subsect. 3.3). We thus conclude that, when the event described in Eq. (66) sets in at time *m*, the state of the system collapses onto one of the states

67 $$\begin{aligned} \omega _{m}^{(r)}(\tilde{Z})=&\langle \Phi _{\sigma ^{m}(\underline{k})}, F\,\, \Phi _{\sigma ^{m}(\underline{k})} \rangle \cdot \text {tr}_{\mathfrak {h}_S}\big (\Omega _{m}^{(r)} \cdot C\big )\,,\,\,\text { where }\nonumber \\ \Omega _{m}^{(r)}:=&\big [\text {tr}_{\mathfrak {h}_S}\big (\Pi _{r}^{(m)}\big )\big ]^{-1}\, \Pi _{r}^{(m)}\,, \,\,\, \text { for some }\,\, r \in \big \{1, \dots , s\big \} . \end{aligned}$$

According to **Axiom CP**, the probability to choose the state $\propto \Pi _{r}^{(m)}$ is given by

$$\begin{aligned} \text {prob}\{r\}= q_{r}^{(m)} \cdot \text {tr}_{\mathfrak {h}_S}\big (\Pi _{r}^{(m)}\big )\qquad (Born's Rule) \end{aligned}$$

Equation (67) implies that, in the *Schrödinger picture*, where “observables” are taken to be *time-independent*, the state, $\Omega _m$, of the atom at time *m* is given by one of the states $\Omega _{m}^{(r)}$.

Equations (63) and (67) complete the induction step, $(\mathcal {I}_{m-1}) \Rightarrow (\mathcal {I}_m)$. $\square$

We note that, when restricted to operators that are functions of the *R*-field, but act trivially on the Hilbert space $\mathfrak {h}_S$ of the atom, the states $\omega _{n},n=0,1,\dots ,$ are *product states*, (the *R*-field has been prepared in the product state $\Phi _{\underline{k}}$). This special choice of a state for the *R*-field implies that the effective time evolution of the state of the atom described above is *“Markovian”*. Moreover, if the atom is *decoupled* from the *R*-field, corresponding to $U=\mathbf {1}$ in Eq. (45), then

$$\begin{aligned} \Omega _n = V^{n}\Omega \, V^{-n}, \qquad \text {with }\,\, \Omega =\widetilde{\Omega } \,\,\text { as in }\,\, (52), \end{aligned}$$

i.e., the evolution of states of the atom is governed by *Schödinger-Liouville evolution* - the atom decoupled from the *R*-field is a *perfectly closed system.*

### A more concrete model of an atom interacting with the *R*-field

*“The concepts ‘system’, ‘apparatus’, ‘environment’, immediately imply an artificial division of the world, and an intention to neglect, or take only schematic account of, the interaction across the split.”* (John Stewart Bell)

It is instructive to study an example of an explicit operator *U* describing interactions between the atom and the *R*-field (see Eq. (45)): We choose a partition of unity, $\big \{Q_m\big \}_{m=1}^{L}\,,$ with  $L\le M$, by orthogonal projections acting on $\mathfrak {h}_S\simeq \mathbb {C}^{M}$ and define *U* by setting

68 $$\begin{aligned} U:= \sum _{m=1}^{L} T^{(m)}\otimes Q_{m}, \end{aligned}$$

where $T^{(m)}$ is a unitary operator on $\mathbb {C}^{N}$, while   $Q_m=Q_m^{*}$ is an orthogonal projection on $\mathfrak {h}_S \simeq \mathbb {C}^{M}$ ,   $Q_m \cdot Q_{\ell } = \delta _{m \ell }\, Q_m, \,\forall \,m, \ell =1,\dots , M, \,\, \text { and }\,\,\sum _{m=1}^{L} Q_m = \mathbf {1}\,.$ For this choice of *U* we find that the Kraus operators are given by

69 $$\begin{aligned} \mathfrak {L}_{\alpha }^{k_{\ell }}= \sum _{m=1}^{L} \langle \phi _{\alpha }, T^{(m)} \phi _{k_{\ell }}\rangle Q_{m}\,. \end{aligned}$$

Let $\Omega _{n-1}$ be the density matrix describing the state of the atom at time $n-1$. In the Schrödinger picture, the state $\widehat{\Omega }_n$ of the atom at time *n*, obtained by restricting $\omega _{n-1}$ to the algebra $\mathcal {E}_{\ge n}$, is then given by Eq. (63) (with $m\rightarrow n$), namely

70 $$\begin{aligned} \widehat{\Omega }_n = \sum _{\ell , m =1, \dots , L} g^{\ell m}(n-1) \, V\,Q_{\ell }\, \Omega _{n-1}\, Q_mV^{*}\,, \end{aligned}$$

where

71 $$\begin{aligned} g^{\ell m}(j):= \langle \phi _{k_j}, (T^{(m)})^{*} \,T^{(\ell )} \phi _{k_j}\rangle = \langle \,T^{(m)}\phi _{k_j}, T^{(\ell )} \phi _{k_j}\rangle \,. \end{aligned}$$

This is a direct consequence of Eqs. (63), (69) and the completeness of $\big \{\phi _{\alpha }\big \}_{\alpha =0}^{N-1}$.

We note that, for an arbitrary time *j*,

72 $$\begin{aligned} \overline{g^{\ell m}(j)}= \langle \, T^{(\ell )} \phi _{k_j} , T^{(m)} \phi _{k_j} \rangle&= g^{m \ell } (j)\,,\quad \quad g^{mm}(j) = 1,\,\,\forall \,m=1,\dots , L,\,\, \text { and }\nonumber \\&\sum _{\ell , m=1}^{L} \overline{v}_{\ell }\, g^{\ell m}(j) \,v_{m} \ge 0\,, \end{aligned}$$

for an arbitrary *L*-tuple, $v:=(v_1,\dots , v_{L})$, of complex numbers; i.e., the matrix

$$\begin{aligned} G(j) := \Big (g^{\ell m}(j)\Big )_{\ell , m= 1}^{L} \end{aligned}$$

is a (hermitian) non-negative matrix on $\mathbb {C}^{L}$ and hence can be diagonalized by a unitary $L\times L$ matrix, $D(j)=\big (d_{r}^{\,s}(j)\big )_{r,s=1}^{L}$:

73 $$\begin{aligned} \gamma _{r}(j)\, \delta ^{rs}= \sum _{\ell m}\,&\overline{d_{\ell }^{\, r}(j)} \, g^{\ell m}(j) \,d_{m}^{\, s}(j)\,,\nonumber \\ i.e., \quad \text {diag}\Big (\gamma (j)&\Big ) = D(j)^{*}\,G(j)\, D(j)\,. \end{aligned}$$

The non-negative numbers $\gamma _{r}(j)$ are the eigenvalues of the matrix *G*(*j*), and we have that

$$\begin{aligned} \sum _{r=1}^{L} \gamma _{r}(j) = \text {tr}\big (G(j)\big ) = \sum _{m=1}^{L}g^{mm}(j) = L\,. \end{aligned}$$

Notice that if $(M\ge ) L > N$ then $L-N$ eigenvalues of *G*(*j*) necessarily vanish. (This is because the vectors $\big \{T^{(\ell )} \phi _{k_j} \,\big |\, \ell =1, \dots L\big \}$ are necessarily linearly dependent if $L>N$.) Physically, it is, however, more realistic to suppose that $N\gg M$. We will see that if one of the eigenvalues $\big \{\gamma _{r}(n-1)\big \}_{r=1}^{L}$ is very close to *L* then the map $\Omega _{n-1} \mapsto \widehat{\Omega }_n$ is close to being given by conjugation with a unitary matrix.

Equation (70) can be cast into the following form: Let $\Omega _{n-1}$ denote the density matrix describing the state of the atom at time $n-1$. Then the density matrix describing the state of the atom at time *n*, *before* **Axiom CP** *is applied*, is given by

74 $$\begin{aligned} \widehat{\Omega }_{n}&= \sum _{r=1}^{L} \gamma _{r}(n-1)\,V \mathfrak {K}_{r}(n-1)\,\Omega _{n-1}\, \mathfrak {K}_{r}(n-1)^{\,*}\,V^{*}\,,\,\,\,\nonumber \\&\quad \text { where }\,\mathfrak {K}_{r}(n-1):= \sum _{m=1}^{L} d_{r}^{\,m}(n-1)Q_{m}\,. \end{aligned}$$

This equation shows that $\widehat{\Omega }_n$ is non-negative, and using that $g^{mm}(n-1) =1,\,\forall \, m=1, \dots , L$, we see that it has trace equal to 1. Thus, $\widehat{\Omega }_{n}$ is again a density matrix, which can be written as a convex combination of disjoint orthogonal projections, $\Pi _{r}^{(n)}$, as in Eq. (65) (with $m\rightarrow n$). Applying **Axiom CP**, we recover an expression equivalent to the one in Eq. (67).

**The weak-coupling regime of the model**

It is interesting to study some limiting regimes in the model introduced above. We first consider the *weak-coupling regime*, which is characterized by

75 $$\begin{aligned} T^{(m)}= \mathbf {1} + \varepsilon \cdot \tau ^{(m)}, \qquad \text { with }\,\, \Vert \tau ^{(m)}\Vert \,\le 1,\,\,\forall \, m=1,\dots , L\,,\,\, \text {and }\,\,0\le \varepsilon \ll 1.\nonumber \\ \end{aligned}$$

It is easy to see that this implies that, for arbitrary *j*,

76 $$\begin{aligned} g^{\ell m}(j) = 1 + \mathcal {O}(\varepsilon ),\qquad \forall \,\, \ell , m\,, \end{aligned}$$

and

$$\begin{aligned} \gamma _{1}(j) = L+\mathcal {O}(\varepsilon )\,, \,\,\, \gamma _{r}(j) =\mathcal {O}(\varepsilon )\,, \,\,\forall \, r>1\,,\,\,\, d_{1}^{\,s}(n) = \frac{1}{\sqrt{L}} \big (1+ \mathcal {O}(\varepsilon )\big )\,, \forall \, s\,. \end{aligned}$$

Equation (70), combined with  $\sum _{m=1}^{L} Q_m =1$, then implies that

77 $$\begin{aligned} \Vert \widehat{\Omega }_{n}- V\, \Omega _{n-1}\, V^{*}\Vert = \mathcal {O}(\varepsilon )\,, \quad i.e.,\, \, \,\, \widehat{\Omega }_n \approx V\,\Omega _{n-1}\,V^{*}\,. \end{aligned}$$

According to the Collapse Postulate,

$$\begin{aligned} \Omega _{n-1}= \mathcal {N}^{-1} \Pi , \,\,\text { where }\,\, \mathcal {N}= \text {tr}_{\mathfrak {h}_S}(\Pi )\,\, \text { and }\,\, \Pi =\Pi ^{*}= \Pi ^{2}\,, \end{aligned}$$

i.e., $\Pi$ is an orthogonal projection. Eqs. (70) and (76) then imply that

78 $$\begin{aligned} \widehat{\Omega }_{n}=&q^{(n)} \Pi ^{(n)} + \sum _{r\ge 2} q_{r}^{(n)} \Pi _{r}^{(n)}, \qquad \text {where }\nonumber \\ q^{(n)}\equiv q_{1}^{(n)} = \mathcal {N}^{-1}+ \mathcal {O}(\varepsilon ),&\quad q_{r}^{(n)} = \mathcal {O}(\varepsilon ),\,\,r\ge 2, \,\, \text { and } \,\, \Vert \Pi ^{(n)} - V\,\Pi V^{*} \Vert = \mathcal {O}(\varepsilon )\,. \end{aligned}$$

The Collapse Postulate (**Axiom CP** of Sect. 3) implies that, with *very high probability*

$$\begin{aligned} \Omega _{n} = \big [\text {tr}_{\mathfrak {h}_S}\big (\Pi ^{(n)}\big )\big ]^{-1} \cdot \Pi ^{(n)} \simeq V\, \Omega _{n-1} V^{*}\,. \end{aligned}$$

Thus, the system obtained by tracing out the *R*-field is well approximated by the closed system consisting of just the atom (decoupled from the *R*-field), whose states evolve unitarily by conjugation with powers of the operator *V*. However, every once in a while, it will happen—for *purely entropic reasons*—that the state of the system collapses onto a very unlikely state $\Omega _{n} \propto \Pi ^{(n)}_{r}$, for some $r\ge 2$, with $\Pi _{r}^{(n)}$ approximately orthogonal to $V\,\Pi \, V^{*}$, which represents a strong deviation from unitary evolution. An observer will perceive a collapse onto such an unlikely state as a *“quantum jump,”* or, put differently, as an *event* in the literal sense of the word. The frequency of collapse onto an unlikely state is proportional to $\varepsilon$.

**The strong-coupling regime of the model**

The strong coupling limit is characterized by the property that

79 $$\begin{aligned} g_{k}^ {\ell m} := \langle T^{(m)} \phi _{k}, T^{(\ell )} \phi _{k} \rangle = \delta ^{\,\ell m} + \mathcal {O}(\varepsilon )\,, \quad \text {with }\,\,\varepsilon \ll 1\,, \end{aligned}$$

for some or all of the vectors $\phi _{k}$, in particular for $\phi _0$. Given a state vector, $\Phi _{\underline{k}}\in \mathfrak {F}_S$, of the *R*-field, we set $g^{\ell m}(j)= g_{k_j}^{\ell m}$, as in Eq. (71). Since, for our choice of a reference vector, $\Phi _{\underline{0}}$, in the construction of the Hilbert space $\mathfrak {F}_S$, we have that $k_j = 0$, except for finitely many values of *j*, the following considerations apply to the analysis of evolution of states at large times under the only assumption that (79) holds for $k =0$. It follows from Eq. (70) that if (79) holds for $k=k_{n-1}$ then

80 $$\begin{aligned} \widehat{\Omega }_n = \sum _{m=1}^{L} V\,Q_m \Omega _{n-1} Q_m \,V^{*}+ \mathfrak {e}_{n}(\varepsilon )\,, \quad \text {with } \,\, \Vert \mathfrak {e}_{n}(\varepsilon ) \Vert = \mathcal {O}(\varepsilon ), \end{aligned}$$

where $\mathfrak {e}_n(\varepsilon )$ is some traceless hermitian $M\times M$ matrix. It then follows from **Axiom CP** of Sect. 3 that $\Omega _n$ is proportional to a spectral projection of $\widehat{\Omega }_n$. We note that if *n* is large enough (depending on the sequence $\underline{k}$) then $k_j =0, \,\forall \, j\ge n-1$, and hence $g^{\ell m}(j) = g_{k_j}^{\ell m} = g_{0}^{\ell m}$ satisfies (79), for all $j \ge n-1$.

#### Remark

The map

81 $$\begin{aligned} \Omega \, \mapsto \, \widehat{\Omega } := \sum _{m =1}^{L} V\,Q_{m}\, \Omega \, Q_{m} \, V^{*}, \end{aligned}$$

is completely positive and trace-preserving; (the operators $\big \{\mathfrak {L}_{m}:=V\,Q_{m}\,\vert \, m=1,\dots , L\big \}$ are Kraus operators).

Next, we consider the following special choice of a partition of unity $\big \{Q_{m}\big \}_{m=1}^{L}$:

82 $$\begin{aligned} Q_m = \vert \psi _m \rangle \langle \psi _m \vert \,, \qquad m=1,\dots , L, \,\text { with }\, L=M=\text {dim}\mathfrak {h}_S\,, \end{aligned}$$

where $\big \{\psi _m\big \}_{m=1}^{M}$ is an orthonormal basis of $\mathfrak {h}_S$. We define a *transition matrix (or -function)*, $P=\big (P(\ell , m)\big )_{\ell , m=1}^{M}$, by setting

83 $$\begin{aligned} P(\ell , m):= \vert \langle \psi _\ell , V \psi _m \rangle \vert ^{2} \ge 0, \qquad \ell , m = 1,\dots , M\,. \end{aligned}$$

The completeness of the vectors $\big \{\psi _m \vert \,m=1,\dots , M\big \}$ and the unitarity of *V* imply that

84 $$\begin{aligned} \sum _{m=1}^{M} P(\ell ,m) = \sum _{\ell =1}^{M} P(\ell , m) =1\,. \end{aligned}$$

Using (80) and (81), we find that, in the strong-coupling regime and for sufficiently large times, the time evolution of the state of the atom in the Schrödinger picture is well approximated by a trajectory of states $\big \{ \psi _{\xi _{n}} \,\vert \, 1\ll n\in \mathbb {Z}_{+}\big \}$ indexed by a **sample path**, $\big \{\xi _{n} \in \mathfrak {X}\,\vert \, n \in \mathbb {Z}_{+} \big \}$, of the Markov chain with state space $\mathfrak {X}:=\big \{1, \dots , M\big \}$ and transition matrix *P* defined in (83). The probabilities

$$\begin{aligned} \mu (m) := \text {prob}\big \{\text {the atom occupies state } \psi _m\big \}, \text { for }\,m=1,\dots , M, \end{aligned}$$

on the state space $\mathfrak {X}$ of the Markov chain evolve approximately according to

85 $$\begin{aligned} \mu _{n}(\ell )= \sum _{m=1}^{M} P(\ell , m)\, \mu _{n-1}(m)\,, \qquad \mu _{n-1}(m)\ge 0, \, \forall \, m, \quad \sum _{m=1}^{M}\mu _{n-1}(m)=1\,,\nonumber \\ \end{aligned}$$

as can be inferred from Eq. (81). The positive number $P(\ell , m)$ can be interpreted as the approximate value of the probability of the event that the atom occupies state $\psi _{\ell }$ at some time *n*, assuming that at time $n-1$ it has occupied state $\psi _{m}$.

Recalling that, in the weak-coupling regime, the Schrödinger-picture time evolution of states of the atom is well approximated by unitary evolution—the one we are used to from text books on elementary Quantum Mechanics—we find that, in the models considered here, the law of evolution of states of the atom in the *ETH*-Approach smoothly interpolates between unitary deterministic Schrödinger evolution, appropriate for closed systems, and classical Markovian evolution of the (state-occupation) probabilities $\mu (\cdot )$, appropriate for isolated open systems of matter very strongly coupled to the radiation field.

**Alternation between unitary evolution and state collapse in measurements**

To conclude this subsection, we sketch how, in suitable situations, the alternation between linear unitary Schrödinger evolution of states of a system and non-linear state collapse in *measurements*, as stipulated in the Copennhagen Interpretation of QM, can be understood as an *approximation* to the fundamental law of evolution of states in the *ETH*-Approach.

We consider models of the kind introduced in Eqs. (68)–(71). Let $U:= \sum _{m=1}^{L} T^{(m)}\,Q_{m}$ , see Eqs. (45) and (68). We decompose the Hilbert space $\mathfrak {h}_S$ of the atom into a direct sum

86 $$\begin{aligned} \mathfrak {h}_S = \mathfrak {h}^{w} \oplus \mathfrak {h}^{s}, \end{aligned}$$

with dim($\mathfrak {h}^{w}) = K < M$, and we assume that the ranges of the projections $Q_1, \dots , Q_J, J\le K,$ are contained in $\mathfrak {h}^{w}$, while the ranges of $Q_{J+1}, \dots Q_L$ are contained in $\mathfrak {h}^{s}$. We interpret the numbers

$$\begin{aligned} g^{\ell m}(j):= \langle \,T^{(m)}\phi _{k_j}, T^{(\ell )} \phi _{k_j}\rangle \end{aligned}$$

as the matrix elements of an $L\times L$ matrix, *G*(*j*) (see below (72)), acting on the vector space

$$\begin{aligned} \mathcal {V}:=\mathbb {C}^{L}= \mathcal {V}^{w} \oplus \mathcal {V}^{s}\,,\,\, \text { where }\,\, \mathcal {V}^{w}\simeq \mathbb {C}^{J},\,\, \text { and }\,\, \mathcal {V}^{s}\simeq \mathbb {C}^{L-J}\,, \end{aligned}$$

$\mathcal {V} \ni \mathbf {v}=(v_1,\dots , v_L) = (\mathbf {v}^{w}, \mathbf {v}^{s})$, with $\mathbf {v}^{w}=(v_1, \dots , v_J)\in \mathcal {V}^{w}$ and $\mathbf {v}^{s}=(v_{J+1}, \dots , v_{L})\in \mathcal {V}^{s}$. The matrix *G*(*j*) is assumed to have the property that

87 $$\begin{aligned} G(j) = G_{0} + \Delta G(j), \qquad G_{0}= G_{0}^{w}\vert _{\mathcal {V}^{w}} \oplus G_{0}^{s}\vert _{\mathcal {V}^{s}}\,, \end{aligned}$$

where

88 $$\begin{aligned} G_{0}^{w} = \begin{pmatrix} 1&{} \ldots &{} 1\\ \vdots &{} &{} \vdots \\ 1&{} \ldots &{}1 \end{pmatrix}\,, \quad G_{0}^{s} = \mathbf {1}\vert _{\mathcal {V}^{s}}\quad \text { and }\quad \Vert \Delta G(j) \Vert \le \varepsilon \ll 1\,. \end{aligned}$$

We also assume that the *time-1* propagator *V* of the atom has the property that

89 $$\begin{aligned} V = V_{0} + \Delta V, \quad \text { where }\,\, V_{0}= V_{0}\vert _{\mathfrak {h}^{w}} \oplus \mathbf {1}\vert _{\mathfrak {h}^{s}}\,, \,\,\,\text {and }\, \,\,\Vert \Delta V \Vert \le \delta \,, \end{aligned}$$

for some $\delta \ll 1$. This implies that it takes a long time of $\mathcal {O}(\delta ^{-1})$ for a state prepared in the subspace $\mathfrak {h}^{w}$ to develop a substantial overlap with a state in the subspace $\mathfrak {h}^{s}$, and the propagator of the atom restricted to the subspace $\mathfrak {h}^{s}$ is very close to the identity operator.

Let us suppose that the initial state of the atom is given by $\Omega _{0}:=\big [\text {tr}(\Pi )\big ]^{-1} \Pi$, where $\Pi$ is an orthogonal projection whose range is contained in $\mathfrak {h}^{w}$, i.e., $\Pi \vert _{\mathfrak {h}^{s}} = 0$, meaning that the initial state of the atom belongs to the subspace $\mathfrak {h}^{w}$ of states only very *weakly* coupled to the *R*-field. Equation (89) then implies that the state of the atom will remain in the subspace $\mathfrak {h}^{w}$ for a period of time of duration $\mathcal {O}(\delta ^{-1})$, with only tiny tails leaking into the subspace $\mathfrak {h}^{s}$. The form of the matrix $G_{0}^{w}$ given in (88) and the fact that $\Vert \Delta G(j) \Vert \le \varepsilon \ll 1$ then entail that the evolution of the state of the atom with initial condition $\Omega _{0}$ is well approximated by unitary Schrödinger evolution, as determined by the *time-1* propagator $V=V_0 +\mathcal {O}(\delta )$ of the atom, for a length of time of $\mathcal {O}(\delta ^{-1})$, until the state of the atom develops a substantial overlap with the subspace $\mathfrak {h}^{s}$. **Axiom CP** of Sect. 3 tells us that, once the state of the atom has a substantial overlap with $\mathfrak {h}^{s}$, it becomes likely that it collapses onto a state, $\Omega ^{s}$, with only a tiny overlap with the subspace $\mathfrak {h}^{w}$. Assumption (88) then implies that the *strong-coupling* law in Eq. (80) governs the further evolution of the state of the atom for a period of time of $\mathcal {O}(\delta ^{-1})$. By assumption (89) the state of the atom then collapses to a projection in the range of one of the projections $Q_m$, with $J+1 \le m\le L$, and stays there for a period of time of duration $\mathcal {O}(\delta ^{-1})$. This can be interpreted as a **measurement** taking place, with a “measurement basis” consisting of the ranges of the projections $Q_{J+1}, \dots , Q_L$.

Our discussion shows that the *time* when the state of the atom collapses from a density matrix whose range belongs to the subspace $\mathfrak {h}^{w}$ of states weakly coupled to the *R*-field to one whose range belongs to the subspace $\mathfrak {h}^{s}$ of states strongly coupled to the *R*-field, signaling the onset of a measurement, is a *random variable*, i.e., it is *not* determined sharply by the theory. Its *distribution/law* is, however, predicted by the theory. In other words, the question *“when does the detector click?”* is answered by saying that the time when it clicks is a random variable whose distribution can be determined by applying the rules of the *ETH*-Approach.

The ideas described here can be incorporated into full-fledged models of measurements performed on micro-systems, such as atoms or molecules, which are only very *weakly* coupled to the *R*-field, but will, through interactions, eventually get entangled with *measuring instruments*, the latter being quantum-mechanical systems *strongly* coupled to the *R*-field (except when they are in their “ground-state”). Explicit examples of models of measurements and measurement instruments will be communicated in a separate paper.

**Other choices of reference states for the** *R* **-field**

To conclude this section we comment on other possible choices of reference vectors used in the construction of the Hilbert space of states of the *R*-field. Of considerable interest are reference vectors exhibiting correlations and entanglement between modes of the *R*-field localized in different time slices. They will be discussed in separate work. Here we consider reference states that can be interpreted as thermal states.

An arbitrary operator in the algebra $\mathcal {A}_{[n,n']}, 0\le n<n',$ is given by a sum of products of operators $A_j$ acting as the identity on all spaces $\mathcal {H}_{\ell }, \ell \not = j,$ and as an $N\times N$ matrix, also denoted by $A_j$, on the space $\mathcal {H}_{j} \simeq \mathbb {C}^{N}$, for $j=n, \dots , n'-1$. An algebra $\mathcal {A}$ is defined by

$$\begin{aligned} \mathcal {A}:= \bigvee _{n<n'<\infty } \mathcal {A}_{[n,n']}\,. \end{aligned}$$

We choose a density matrix, $\Phi$, on $\mathbb {C}^{N}$ by setting

90 $$\begin{aligned} \Phi = \sum _{k=1}^{K} p_{k} \,\big [\text {tr}(P_{k})\big ]^{-1} P_{k} \,, \quad 1\ge p_1> \dots> p_K > 0\,, \quad \sum _{k=1}^{K} p_{k} =1\,, \,\,\, K\le N\,,\nonumber \\ \end{aligned}$$

where $\big \{P_{k}\big \}_{k=1}^{K}$ is a family of orthogonal projections on $\mathbb {C}^{N}$, with $P_{k}\cdot P_{\ell }=\delta _{k \ell } P_{k}\,,\, \forall \, k, \ell$, and $\sum _{k} P_{k} \le \mathbf {1}\vert _{\mathbb {C}^{N}}$. For later purposes, we set $P_0 := \mathbf {1}\vert _{\mathbb {C}^{N}}$. Physically, the density matrix $\Phi$ may describe a thermal state of the *R*-field in a time slice.

We define a product state $\varphi$ on $\mathcal {A}$ by setting

91 $$\begin{aligned} \varphi (A)= \prod _{j=n}^{n'} \text {tr}\big (\Phi \cdot A_j\big ), \quad \text { for }\,\, \,A=\bigotimes _{j=n}^{n'}A_j \in \mathcal {A}_{[n,n']}\subset \mathcal {A}\,, \end{aligned}$$

with $\Phi$ as in (6,90).

A Hilbert space, $\mathfrak {F}_{\varphi }$, of state vectors of the *R*-field is obtained by applying the so-called *GNS construction* to the pair $\big (\mathcal {A}, \varphi \big )$ (see, e.g., [25]). The space $\mathfrak {F}_{\varphi }$ carries a $^{*}$representation, $\pi _{\varphi }$, of $\mathcal {A}$; in the following, we will not distinguish between *A* and $\pi _{\varphi }(A)$, for $A\in \mathcal {A}$. For an operator $A=\bigotimes _{j=n}^{n'}A_j,\, \text { with }\,\,A_j \in B(\mathcal {H}_j),$ we define

92 $$\begin{aligned} \sigma (A):= \bigotimes _{j=n}^{n'} A_{j}\vert _{\mathcal {H}_{j+1}}\,. \end{aligned}$$

This defines a $^{*}$automorphism of the algebra $\mathcal {A}$. It is obvious that the state $\varphi$ is invariant under $\sigma$, i.e.,

$$\begin{aligned} \varphi \big (\sigma (A)\big )=\varphi (A), \quad \forall A\in \mathcal {A}\,, \end{aligned}$$

which implies that there is a unitary operator $\mathfrak {S}$ acting on $\mathfrak {F}_{\varphi }$ such that

93 $$\begin{aligned} \sigma (A)= \mathfrak {S}^{-1} \,A\,\mathfrak {S}\,, \quad \forall \, A\in \mathcal {A}\,. \end{aligned}$$

The operator $\mathfrak {S}$ generates a unitary propagator on $\mathfrak {F}_{\varphi }$ for the *R*-field in the Schrödinger picture.

We introduce event algebras

$$\begin{aligned} \mathcal {A}_{\ge n}:= \overline{\bigvee _{n' >n} \pi _{\varphi }\big (\mathcal {A}_{[n,n']}}\big )\,, \end{aligned}$$

where the closure is taken in the topology of weak convergence of operators on $\mathfrak {F}_{\varphi }$.

As before, we monitor the evolution of the system only for times $n\ge 0$. In order to find the explicit law of the time evolution of states predicted by the *ETH*-Approach (see Definition 6 and **Axiom CP**, Sect. 3), we have to determine the center, $\mathcal {Z}_{\varphi }(\mathcal {A}_{\ge n})$, of the centralizer of the state $\varphi$ restricted to the algebra $\mathcal {A}_{\ge n}$, for an arbitrary $n\ge 0$. Since $\varphi$ is a time-translation invariant product state, the value of *n* is unimportant. Among orthogonal projections belonging to $\mathcal {Z}_{\varphi }(\mathcal {A}_{\ge n})$ are all the operators

94 $$\begin{aligned} \pi _{\underline{k}} := \bigotimes _{j\ge n} P_{k_j}\vert _{\mathcal {H}_j}, \quad \text { with }\,\,\underline{k}\in \mathcal {S}_{fin}\,, \end{aligned}$$

where $\mathcal {S}_{fin}$ is the set of sequences $\underline{k}=\big \{k_j\big \}_{j=0}^{\infty }$ with the property that $k_j =0$, except for finitely many $j\in \mathbb {Z}_{+}$. It is then not difficult to see that the spectrum, $\mathfrak {X}$, of $\mathcal {Z}_{\varphi }(\mathcal {A}_{\ge n})$ is **continuous**; (it is homeomorphic to the interval [0, 1]). This is a new feature exhibited by the model considered here, which motivates one to generalize the collapse postulate, **Axiom CP** of Sect. 3, to systems featuring actual events,

$$\begin{aligned} \big \{ \pi _{\xi }\,\vert \,\xi \in \mathfrak {X} \big \} \,\,\text {generating }\,\, \mathcal {Z}_{\omega _{\,t}}\big (\mathcal {E}_{\ge t}\big ), \end{aligned}$$

(see Definition 6 of Sect. 3) with a spectrum $\mathfrak {X}$ that can be a *continuous* topological (compact Hausdorff) space. An extension of our theory to this situation will be pursued elsewhere.

We observe that, in the models considered in this section, an arbitrary projection $\pi ^{(n)}$ in $\mathcal {Z}_{\varphi }(\mathcal {A}_{\ge n})$ has the form

$$\begin{aligned} \pi ^{(n)} = P\vert _{\mathcal {H}_n} \otimes \pi _{\ge (n+1)}, \end{aligned}$$

where *P* is a spectral projection of the density matrix $\Phi \vert _{\mathcal {H}_n}$ and $\pi _{\ge (n+1)} \in \mathcal {A}_{\ge (n+1)}$.

If $P= P_{k_1}+\dots + P_{k_J},$ for some  $1< J\le K,$ where the operators $P_{k_j}$ are spectral projections of $\Phi$, then $\pi ^{(n)}$ can be decomposed into a non-trivial sum of projections,

$$\begin{aligned} \pi ^{(n)}= \sum _{j=1}^{J} P_{k_j}\otimes \pi _{\ge (n+1)}\,. \end{aligned}$$

Next, we recall the Collapse Postulate, **Axiom CP**, in Sect. 3. In the context of the models discussed in this section it is natural to generalize this postulate as follows: If $\varphi$ is the initial state of the *R*-field then the state, $\varphi _n$, on the algebra $\mathcal {A}_{\ge n}$ visited, at time *n*, along some history of the system with initial condition $\varphi$ for the *R*-field has the form

95 $$\begin{aligned} \varphi _{n} = P_{k_n} \otimes \varphi ^{(n+1)}\,, \quad \, \text { for some }\,\, k_n = 1,\dots K\,, \end{aligned}$$

where   $\varphi ^{(n+1)}$   is a normal state on   $\mathcal {A}_{\ge (n+1)}$. The frequency of choosing $k_n=k_{*}$, for some $k_{*}\in \{1,\dots , K\}$, is given by

96 $$\begin{aligned} \text {prob}(k_n=k_{*}) = p_{k_{*}}, \quad \text { with }\,\, \,p_{k_{*}} \,\, \text { as in }\,\, \, \text {Eq.}\,(90)\,. \end{aligned}$$

In the Schrödinger picture, the time evolution of the state of the atom coupled to the *R*-field predicted by the *ETH*-Approach (see Definition 6 and **Axiom CP** of Subsect. 3.3) is then described by Eq. (70), where the coefficients $g^{\ell m}$ are given by

97 $$\begin{aligned} g^{\ell m}(j):= \big [\text {tr}(P_{k_j})\big ]^{-1} \text {tr}\big (\, T^{(\ell )}\,P_{k_j}\, (T^{(m)})^{*} \big )\,. \end{aligned}$$

These coefficients are *random variables* whose law is given by (96).

It is clear that properties (95), (96) and (97) hold for sufficiently large times, *n*, for a subspace of initial states of the *R*-field dense in $\mathfrak {F}_{\varphi }$. It would be interesting to analyze properties of the dynamics of the atom, with the *randomness* in the time evolution of its states caused by the repeated collapse of the state of the *R*-field, as described in Eqs. (95) and (96).

## Conclusions and Outlook

“*The interpretation of quantum mechanics has been dealt with by many authors, and I do not want to discuss it here. I want to deal with more fundamental things.”* (Paul Adrien Maurice Dirac)

Our main goal in this paper has been to illustrate the *ETH* **-Approach to Quantum Mechanics**, which many readers may find rather abstract, with a discussion of simple, concrete models, which are, however, sophisticated enough to exhibit some of the main subtleties and virtues of the *ETH*-Approach. The models used in Sect. 5 to illustrate the general ideas underlying this approach to Quantum Mechanics have been inspired by Huygens’ Principle in quantum field theory and, in particular, by the form it takes in the limit where the speed of light tends to $\infty$; see Sect. 4. The main results of our analysis are contained in Sects. 3 and 5 .

To conclude this paper, we attempt to clarify what we consider to be the *ontology* underlying Quantum Mechanics, as suggested by the *ETH*-Approach. We then present some comments on models arising from those in Sect. 5 by letting the time step approach 0, i.e., when choosing time to be a continuous parameter. Finally, we offer a sketchy discussion of different mechanisms that might give rise to the *Principle of Diminishing Potentialities* and a survey of some important open problems.

### From ‘what may potentially be’ to ‘what actually *is*’

“*The Garden of Forking Paths is a picture, incomplete yet not false, of the universe.”* (Jorge Luis Borges)

The summary of the *ETH*-Approach presented in Sect. 3 and the discussion of concrete models contained in Sect. 5 provide a fairly clear idea of what might be considered to be the *ontology* underlying Quantum Mechanics. In order to keep the following remarks as accessible as possible, we shall discuss this topic in the context of the models studied in the last section.

Equation (47) of Sect. 5 shows that, in the (idealized) models of physical systems studied there, the event algebras $\mathcal {E}_{\ge n}, n\ge 0,$ are all unitarily equivalent to one “universal” algebra $\mathcal {N}\equiv \mathcal {E} := \mathcal {A}_{\ge 0}\otimes B(\mathfrak {h}_S)$. (Recall that we only monitor the evolution of the systems for times $t\ge t_{in}=0$.) The fact that $\mathcal {E}_{\ge n} \simeq \mathcal {E}, \forall \, n\ge 0,$ enables us to define the *non-commutative spectrum*, $\mathfrak {Z}_S$, of the systems described by our models by setting

$$\begin{aligned} \mathfrak {Z}_{S}:= \bigcup _{\omega } \Big (\,\omega , \mathcal {Z}_{\omega }(\mathcal {E})\Big )\,, \end{aligned}$$

where the union is a disjoint union ranging over all normal states $\omega$ on the algebra $\mathcal {E}$, and $\mathcal {Z}_{\omega }(\mathcal {E})$ is the center of the centralizer of the state $\omega$ restricted to the algebra $\mathcal {E}$; see Eq. (20) of Sect. 3. The algebra $\mathcal {Z}_{\omega }(\mathcal {E})$ is abelian. Its projections provide a mathematical description of the actual event featured by the system when it occupies the state $\omega$.

Let $\gamma$ denote the $^{*}$-endomorphism of the algebra $\mathcal {E}$ corresponding to time translation of operators in the Heisenberg picture by a time step of length 1; i.e.,

$$\begin{aligned} \mathcal {E}_{\ge 1} \equiv \gamma (\mathcal {E}) \subset \mathcal {E}\,. \end{aligned}$$

#### Remark

If time translations are unitarily implementable on the Hilbert space, $\mathcal {H}_S$, of state vectors of the system *S* then one has that $\gamma (X)= \Gamma ^{-1} X\, \Gamma , \,\, \forall \,\, X\in \mathcal {E}$, where $\Gamma$ is the unitary propagator on $\mathcal {H}_S$ by a time step of length 1; see Sects. 3 and 5 .

Given the algebra $\mathcal {E}$ and the time-translation endomorphism $\gamma$ on $\mathcal {E}$, the space of normal states on $\mathcal {E}$ can be equipped with the structure of a *groupoid*:

For a given pair, $(\omega , \omega ')$, of normal states on $\mathcal {E}$, there is an *arrow* from $\omega$ to $\omega '$, written as   $\omega \rightarrow \omega '$, iff there exists a minimal orthogonal projection $\pi \in \mathcal {Z}_{\omega }(\mathcal {E}_{\ge 1})$ (i.e., $\pi$ cannot be decomposed into a sum of two or more non-zero projections belonging to $\mathcal {Z}_{\omega }(\mathcal {E}_{\ge 1})$) such that

98 $$\begin{aligned} \omega (\pi )>0,\quad \text { and }\quad \omega '\big (X\big ) = \big [\omega (\pi )\big ]^{-1} \omega \big (\pi \,\gamma (X)\, \pi \big )\,, \qquad \forall \,\, X\in \mathcal {E}\,. \end{aligned}$$

#### Definition 9

A *history* of length *r* is a connected path, $\underline{\omega }_{r}:= \big (\omega _0, \dots , \omega _{r}\big )\,,$ of states on $\mathcal {E}$, with the property that

99 $$\begin{aligned} \omega _j \rightarrow \omega _{j+1}\,, \, \, \,\forall \,\,j=0, \dots , r-1\,. \end{aligned}$$

$$\square $$

If $\underline{\omega }_{r}$ is a history of length *r* then there exist minimal orthogonal projections $\pi _j \in \mathcal {Z}_{\omega _j}(\mathcal {E}_{\ge 1})$, with $\omega _{j}(\pi _j)>0, \text { for }\, j=0, \dots , r-1$, such that

100 $$\begin{aligned} \omega _{j+1}(X)= \big [\omega _{j}(\pi _j)\big ]^{-1} \omega _{j}(\pi _j \, \gamma (X)\, \pi _j)\,, \quad \forall \,\, X\in \mathcal {E}\,,\,\forall \,\,j=0,\dots , r-1\,.\nonumber \\ \end{aligned}$$

Thus, a history $\underline{\omega }_r$ of length *r* can also be parametrized by a pair $\big (\omega , \underline{\pi }_r \big )$, where $\omega =\omega _0$ is the inital state of the system, and the sequence of projections, $\underline{\pi }_r =\big (\pi _0, \dots , \pi _{r-1}\big )$ is such that Eq. (100) holds. The space of histories with initial condition $\omega \equiv \omega _0$ is denoted by $\mathfrak {H}_{\omega }$. We define *history operators*

101 $$\begin{aligned} H(\underline{\pi }_r):= \prod _{j=0}^{r-1}\gamma ^{j}(\pi _j)\,, \quad \underline{\pi }_r =\big (\pi _0, \dots , \pi _{r-1}\big )\,,\quad r=1,2,3,\dots \end{aligned}$$

History operators can be used to equip $\mathfrak {H}_{\omega }$ with a probability measure, $\mathbb {P}_{\omega }$:

102 $$\begin{aligned} \mathbb {P}_{\omega }\big (\underline{\pi }_r\big ):= \omega \big ( H(\underline{\pi }_r)^{*}\cdot H(\underline{\pi }_r)\big )\,,\quad \omega =\omega _{0} \,, \end{aligned}$$

with $\pi _{j} \in \mathcal {Z}_{\omega _j}(\mathcal {E}_{\ge 1})$ and $\omega _j$ as in (100), for $j=0, \dots , r-1$. We have that

$$\begin{aligned} \sum _{\pi _{r-1} \in \mathcal {Z}_{\omega _{r-1}}(\mathcal {E}_{\ge 1})} \mathbb {P}_{\omega }\big (\underline{\pi }_r\big ) = \mathbb {P}_{\omega }\big (\underline{\pi }_{r-1}\big )\,, \end{aligned}$$

which follows readily from the definition of history operators, the fact that $\pi ^{2}=\pi = \pi ^{*}$, for an arbitrary orthogonal projection, and from the property that the projections $\pi _{r-1}\in \mathcal {Z}_{\omega _{r-1}}(\mathcal {E}_{\ge 1})$ form a partition of unity. *Kolmogorov*’s extension lemma then tells us that $\mathbb {P}_{\omega }$ extends to a probability measure on the space $\mathfrak {H}_{\omega }$ of infinite histories with initial condition $\omega$.

Formula (102) is reminiscent of the *Lüders-Schwinger-Wigner* formula [38–40] for the probability of outcomes of repeated measurements; but it has an entirely different, logically satisfactory status and interpretation.

In the *ETH*-Approach, the **ontology of Quantum Mechanics** lies in the **histories** traversed by isolated physical systems. Put differently, one might say that what really **“exists”** is encoded into sequences

$$\begin{aligned} \Big \{ \Big (\omega _j, \mathcal {Z}_{\omega _j}(\mathcal {E}) \Big ) \,\Big | \, \omega _j \rightarrow \omega _{j+1},\,\text {for }\, j=0, \dots , r-1\Big \}, \quad r=1,2,3,\dots \,, \end{aligned}$$

of pairs of states and actual events, with Eq. (100) providing the relation between $\omega _j, \pi _j$ and $\omega _{j+1}$.

We note that a state $\omega$ on the algebra $\mathcal {E}$ gives rise to an actual **E**vent described by $\mathcal {Z}_{\omega }(\mathcal {E})$; the space of normal states on $\mathcal {E}$, viewed as a groupoid with an arrow defined in Eq. (100) that connects them to a fixed initial state $\omega =\omega _0$, has a **T**ree-like structure; and the states occupied by the system in the course of time form a **H**istory, i.e., an element of the space $\mathfrak {H}_{\omega }$ of histories of the system starting with the state $\omega$. This explains why the formulation of Quantum Mechanics explored in this paper is called **ETH**-Approach.

*Problem* Generalize the theory developed in Sect. 3 and exemplified by the models in Sect. 5 to apply to physical systems with the following properties:

- They have states, $\omega$, of physical interest that give rise to centers, $\mathcal {Z}_{\omega }(\mathcal {E})$, of centralizers with *continuous spectrum*. (A preliminary version of such a generalization has been worked out and will appear elsewhere.)
- Time is continuous, $t\in \mathbb {R}$.
- They are described by some *relativistic local quantum theory*; (with ‘time’ traded for ‘space-time,’ $t\in \mathbb {R} \mapsto P\in \mathbb {M}^{4}$). A beginning of such a theory has been outlined in [13].

We expect that the first problem stated here can be solved without major difficulties. Comments on the second and third problem follow in the next subsections.

### Models with continuous time

*“Time does not pass, it continues.”* (Marty Rubin)

Recall the family of models discussed in Sect. 5. One should ask whether these models remain meaningful in the limit where the time step tends to 0, i.e., for a continuous time parameter. To answer this question, we consider an *R*-field defined in terms of its $\mathbb {M}_{N}(\mathbb {C})$-valued creation- and annihilation operators, $a^{*}(t)$ and *a*(*t*), with

103 $$\begin{aligned} a^{\#} = a\,&\text { or }\, a^{*}, \qquad a^{\#}(t)= \big (a_{ij}^{\#}(t)\big )_{i,j=1,\dots , N}, \,\text { and }\nonumber \\ \big [a_{ij}^{\#}(t), a_{k \ell }^{\#}(t')\big ] =0,\qquad&\big [a_{ij}(t), a_{k\ell }^{*}(t')\big ]= \delta _{ik}\,\delta _{j\ell }\cdot \delta (t-t')\,, \quad \forall \,\, i,j,k, \ell , \quad \forall \,\,t \,, t' \text { in }\mathbb {R}\,. \end{aligned}$$

Let $\mathfrak {F}$ denote the Fock space corresponding to these creation- and annihilation operators; the creation- and annihilation operators, $a_{ij}^{\#}(\cdot ),$ being operator-valued distributions on $\mathfrak {F}$. Fock space contains a vector $\vert 0 \rangle$, called *vacuum vector*, with the property

$$\begin{aligned} a_{ij}(t)\vert 0 \rangle = 0, \qquad \forall \,\, i,j = 1,\dots , N, \,\, \forall \,\, t\in \mathbb {R}\,. \end{aligned}$$

Applying arbitrary polynomials in creation operators, smeared out with $\mathbb {M}_{N}(\mathbb {C})$-valued test functions on the time axis $\mathbb {R}$, to the vacuum vector $\vert 0\rangle$ generates a dense set of vectors in $\mathfrak {F}$. Fourier transformation in the variable *t* yields creation- and annihilation operators, $\hat{a}^{*}(\nu )$ and $\hat{a}(\nu )$, related to $a^{*}(t)$ and *a*(*t*) by

$$\begin{aligned} a_{ij}^{\#}(t)= \int _{\mathbb {R}} d\nu \,e^{\pm i (t\cdot \nu /2\pi )}\, \hat{a}_{ij}^{\#}(\nu ), \end{aligned}$$

and satisfying the commutation relations (103), with time *t* replaced by frequency $\nu$. Time translations on $\mathfrak {F}$ are generated by an operator

104 $$\begin{aligned} H_R := \frac{1}{2\pi } \sum _{i.j=1}^{N}\int _{\mathbb {R}} d\nu \, \hat{a}_{ij}^{*}(\nu )\, \nu \,\hat{a}_{ij}(\nu )\,. \end{aligned}$$

The Hamilton operator $H_R$ is self-adjoint on a natural dense subspace of $\mathfrak {F}$ and generates unitary time translations,

$$\begin{aligned} \Gamma _t = e^{-it\,H_R}, \quad t\in \mathbb {R}, \end{aligned}$$

on $\mathfrak {F}$. We observe that the spectrum of $H_R$ covers the entire real axis,^19^ that the vacuum vector $\vert 0\rangle$ is invariant under time-translations, i.e., $e^{itH_R}\vert 0 \rangle = \vert 0 \rangle ,\, \forall \, t$, and that it is a product state. This last property follows from the form of the two-point function,

$$\begin{aligned} \langle 0\vert a_{ij}(t)\, a^{*}_{k\ell }(t')\vert 0\rangle = \delta _{ik}\delta _{j\ell } \delta (t-t')\,, \end{aligned}$$

and Wick’s theorem (see, e.g., [34]).

Next, we add the “atom” to the play and introduce interactions between the atom and the *R*-field. We continue to assume that the atomic Hilbert space is finite-dimensional, $\mathfrak {h}_S\simeq \mathbb {C}^{M},$ for some $M< \infty$. The Hilbert space for the atom coupled to the *R*-field is given by $\mathcal {H}_S = \mathfrak {F}\otimes \mathfrak {h}_S$. Before it is coupled to the *R*-field the propagator of the atom is generated by a hermitian matrix, $H_A$, on $\mathfrak {h}_S$. The interaction between the atom and the *R*-field is specified by a self-adjoint (bounded) operator, $W=W^{*}$, on $\mathcal {H}_S$. The total Hamiltonian of the system *S* is then given by

105 $$\begin{aligned} H:= H_R\otimes \mathbf {1} + \mathbf {1}\otimes H_A + W \end{aligned}$$

The limit of continuous time of the model studied in Subsect. 6.4 corresponds to the choice

$$\begin{aligned} W= \sum _{m=1}^{L} v_m\otimes Q_m\,, \end{aligned}$$

where $\big \{Q_m\big \}_{m=1}^{L}$ is a partition of unity on $\mathfrak {h}_S$ by orthogonal projections, as in Subsect 5.4, and the operators $v_m$ are self-adjoint bounded operators on $\mathfrak {F}$, (with $e^{-iv_m} = T^{(m)}$).

Models of this sort have been studied in the literature; see, e.g., [41, 42] and refs. given there.^20^ Before the Collapse Postulate, **Axiom CP** of Sect. 3, is imposed the effective time evolution of the atomic degrees of freedom is given by a Lindbladian evolution [44, 45]. In [46], the authors derive a non-linear stochastic Schrödinger equation for the state vector—the state of the atom in our model—from Lindbladian evolution of the density matrix when the Collapse Postulate is imposed. Their results can be applied to the model introduced above.

One might argue that one should attempt to derive continuous-time limits of the more natural (semi-relativistic) models studied in Sect. 4, which could be expected to have Hamiltonians that are bounded from below. However, this project is obstructed by our inability to construct models of *local relativistic quantum theory*, in particular Quantum Electrodynamics, without ultraviolet cutoffs. Thus, in the realm of (semi-)relativistic models of atoms coupled to the quantized electromagnetic field satisfying the Principle of Diminishing Potentialities, we may be stuck with models that have a discrete time, as discussed in Sect. 4. (See, however, [13] for an “axiomatic” analysis of the *ETH*-Approach in the context of local relativistic quantum theory.)

### Are there alternatives to Huygens’ principle in deriving the principle of diminishing potentialities?

*“One finds in this subject a kind of demonstration which does not carry with it so high a degree of certainty as that employed in geometry,...”* (Christiaan Huygens)

In this last subsection, we draw the readers’ attention to the problem to identify physical mechanisms that give rise to the *Principle of Diminshing Potentialities* (*PDP*) (see Eq. (14), Sect. 3). We have seen in Sect. 4 that (*PDP*) is implied by *Huygens’ Principle* in local relativistic quantum theories involving *massless modes* (see also [20]) and by the form this principle takes in quantum theories obtained in the limit of the speed of light tending to $\infty$. This suggests to study the question on what space-times Huygens’ Principle is known to be valid.

- Huygens’ Principle is known to hold in theories on Minkowski space-times, $\mathbb {M}^{d}$, of *even* dimension, i.e., for even *d*; and it is known to fail in theories on odd-dimensional Minkowski space-times.
- Huygens’ Principle holds on *even-dimensional* space-times diffeomorphic to (a half-space contained in) $\mathbb {M}^{d}$, with a metric that differs from the standard Lorentzian metric on $\mathbb {M}^{d}$ only by a *conformal factor*. An example is the spatially flat Friedman-Lemaître universe.
  It would be of interest to compile a list of space-times on which Huygens’ Principle holds true.
- We expect that (*PDP*) holds on certain even-dimensional space-times with black holes. But we have not studied this issue in any detail, yet.
- There are even-dimensional space-time manifolds with non-vanishing curvature on which Huygens’ Principle *fails*. However, this may not invalidate (*PDP*), as remarked next.
- Huygens’ Principle and (*PDP*) could hold if it turned out that “visible” space-time is a submanifold of positive co-dimension of a space-time manifold with *extra dimensions*, and only certain massless modes could and would penetrate into the bulk of the higher-dimensional space-time manifold (*even* if, on the submanifold corresponding to the “visible” space-time, Huygens’ Principle might fail).

The Principle of Diminishing Potentialities constrains the inclusions of algebras generated by *potential events/potentialities* localized in the future of different causally ordered points in space-time; see Sect. 4 and [13]. If gravity is neglected it is clear what is meant by the future of a space-time point *P*: It is the future light cone, $V^{+}_P$, erected over *P*, and the potentialities localized in the future of *P* are certain operators localized in $V^{+}_P$ that generate an algebra denoted by $\mathcal {E}_{\ge P}$. In a local relativistic quantum theory with massless particles on an even-dimensional Minkowski space satisfying Huygens’ Principle, such as quantum electrodynamics, we then have that $\mathcal {E}_{\ge P'} \subsetneq \mathcal {E}_{\ge P}$, whenever the space-time point $P'$ lies in the future of *P*; see [20]. This yields a form of the Principle of Diminishing Potentialities well suited for such theories, as argued in [13]. The bundle of future light cones over space-time is determined by the conformal structure of space-time, and Huygens’ Principle is tied to properties of the propagation of (massless) waves on space-time.

However, if gravitational effects are taken into account the structure of future light-cones in space-time and the metric in the vicinity of future light-cones are not determined a priori, because quantum theory does never determine with certainty *what events/actualities* will happen. Since events couple to gravity, the metric structure of the “future” is not determined a priori. For these reasons, the Principle of Diminishing Potentialities should really be formulated in a way that does *not* depend on knowledge of the conformal structure in the vicinity of future light-cones in space-time. One should look for a more abstract, *“background-independent”* formulation of (*PDP*), one that incorporates gravitational effects.

We recall that one expects that, in a given local relativistic quantum theory, all event algebras, $\mathcal {E}_{\ge P}$, associated with the future above an arbitrary space-time point *P* are isomorphic to a universal algebra $\mathcal {N}$. The Principle of Diminishing Potentialities can then be seen as a consequence of the existence of one-parameter semi-groups, $\big \{\gamma _t\big \}_{t\in [0, t_{*})}\,, 0< t_{*} \le \infty$, of $^{*}$-endomorphisms of $\mathcal {N}$ with the property that

106 $$\begin{aligned} \gamma _{t}\big (\mathcal {N}\big ) \subsetneqq \mathcal {N}, \qquad \forall \,\, 0< t < t_{*}\,. \end{aligned}$$

The fundamental problem is to come up with a general characterization of algebras that can play the role of $\mathcal {N}$ and of one-parameter semi-groups $\big \{\gamma _t\big \}_{t\in [0, t_{*})}$ on such algebras satisfying (106). This problem appears to be a very difficult one.

Returning to quantum theories on Minkowski space, with gravity neglected, our analysis leads to the following somewhat tantalizing general **conjecture**: If we consider a quantum theory for a system *S* in which (*PDP*) holds and with a Hamiltonian, *H*, generating Heisenberg-picture time translations of operators representing physical quantities of *S* that satisfies the spectrum condition, i.e., $H\ge 0$, then this theory must necessarily be a *local relativistic* quantum theory on an even-dimensional Minkowski space. In other words, a quantum theory describing *events* and *measurements*, which does not have states of arbitrarily negative energy, must be a local relativistic theory on an even-dimensional space-time.

To conclude this discussion, one might say that the Principle of Diminishing Potentialities (*PDP*) is really the appropriate general formulation of Huygens’ Principle in local quantum theory. There may not be any viable alternatives to (*PDP*) if we want quantum theory to describe events (including measurements and observations). Thus, a clarification of the status of the Principle of Diminishing Potentialities may be viewed as a fundamental problem of Quantum Physics.

### An agenda for the future

We end this paper by summarizing some important-looking problems.

1. Investigate the time evolution of states determined by the rules of the *ETH*-Approach in more realistic models of simple isolated systems featuring events, in the spirit of the analysis presented in Sects. 4 and 5 . Models with continuous time (in the limit where the speed of light approaches $\infty$) and models describing measurements of physical quantities of small subsystems coupled to quantum-mechanical measuring devices will be of particular interest.
2. Pursue a general study of local relativistic quantum theories on even-dimensional Minkowski spaces, extending the approach outlined in [13]. An important question to be answered is whether the space-time localization of projections describing actual events can be determined more precisely than has been possible, so far. Some ideas discussed in [47] may be relevant in this connection.
3. Explore the relation between the *Principle of Diminishing Potentialities* and the *(quantum) structure of space-time* on large and on very short distance scales, beyond the results presented in [20] and in this paper.

## Footnotes

1 With a clear ontological meaning.

2 During the Song Dynasty, Xú Zi Píng reformed Li Xu-Zhong’s *“Three Pillars of Destiny”* by adding the *birth time* as the *“fourth pillar.”...* Source: wikipedia.org/wiki/Four Pillars of Destiny.

3 i.e., conjugation of operators representing physical quantities by the unitary propagator of the system.

4 We regard it as an interesting and rather fundamental problem of quantum physics to better understand this connection in a general context and to explore its relations to the structure of space-time, as well as to study alternative foundations of the *Principle of Diminishing Potentialities*.

5 We acknowledge that many of the quotations in this paper are taken from Wikipedia.

6 In order to comprehend the notion of physical quantities underlying the analysis presented in this paper, the reader may find it useful to recall the description of, for example, a quantum gas, such as the electon gas in a metal or a gas of bosonic atoms, in the *formalism of second quantization*. See also Sect. 5.

7 If a notion of “state” is supposed to have concrete physical (ontological) meaning then a “state” should be a functional that is only evaluated on operators describing “future potentialities” (rather than on arbitrary operators acting on $\mathcal {H}_S$); see Sect. 3.

8 This would not be too serious a problem if all we intended to do is to describe a *single* measurement (at the “end of time”), but gave up the requirement that the theory ought to also consistently describe *repeated* measurements.

9 *“[The probability wave] ... introduced something standing in the middle between the idea of an event and the actual event, a strange kind of physical reality just in the middle between possibility and reality.”* (Werner Heisenberg).

10 Operators representing physical quantities do not play an important role in the following analysis, anymore. The question how events can be monitored by measuring physical quantities has been studied in detail in [11, 13].

11 In earlier work, see [10], (*PDP*) has been called *Loss of Access to Information (LAI)*.

12 Our criterion is inspired by the desire to rescue as many of the more reasonable features of the *Copenhagen interpretation* of quantum mechanics as possible.

13 The algebra $\mathcal {Z}_{\omega _{t}}(\mathcal {E}_{\ge t})$ is an *abelian* von Neumann algebra. On a separable Hilbert space, it is generated by a single self-adjoint operator *X* (an “observable”), whose spectral projections yield the projections $\lbrace \pi _{\xi }\,\vert \, \xi \in \mathfrak {X} \rbrace$ describing a potential event. This is the motivation behind (16). For simplicity, we do not consider operators, *X*, with continuous spectrum, and the spectra $\mathfrak {X}$ are assumed to be discrete. Generalizations will be considered elsewhere.

14 This is the case in the models considered in Sect. 5 and in relativistic Quantum Electrodynamics [20].

15 “States of physical interest” of a physical system are those normal states the system can actually be prepared in. Here we leave this notion a little vague; but see [23, 33].

16 An unbounded self-adjoint operator *A* is affiliated with a von Neumann algebra $\mathfrak {M}$ if and only if all bounded functions (spectral projections) of *A* belong to $\mathfrak {M}$.

17 Actually, these algebras are von Neumann algebras (factors) of type $III_{1}$; see [20, 25].

18 It is easy to extend our arguments to models with $\mathcal {H}_n$ infinite-dimensional; but this does not render the analysis more interesting.

19 It is a general theorem that the existence of *time-translation invariant product states* implies that the spectrum of the Hamiltonian is unbounded from above and from below.

20 They have also come up in connection with the problem of *time* in QM and quantum systems describing *clocks*; see [43]. The literature on this topic is somewhat hard to decipher.
